# CTRP9 as a myokine mitigates sarcopenia via the LAMP-2A/NLRP3 pathway

**DOI:** 10.1038/s41419-025-08025-w

**Published:** 2025-10-07

**Authors:** Linxi Li, Anju Zuo, Ruoyu Yin, Qiangqiang Liu, Chen Liu, Na Li, Dan Xu, Shaomeng Zhang, Jiarui Li, Shengyun Lei, Shiyan Ruan, Tingting Li, Yuan Guo

**Affiliations:** 1 Department of General Practice, Qilu Hospital of Shandong University, Jinan, Shandong China; 2 The Key Laboratory of Cardiovascular Remodeling and Function Research, Chinese Ministry of Education, Chinese Ministry of Health, Qilu Hospital of Shandong University, Jinan, China; 3 Department of General Practice, Shulan Hospital, Jinan, Shandong China; 4 Department of Internal Medicine, Jinan Maternity and Child Care Hospital Affiliated to Shandong First Medical University, Jinan, China; 5 Department of Pulmonary and Critical Care Medicine, Qilu Hospital of Shandong University, Jinan, Shandong China; 6 Department of Ultrasound, Qilu Hospital of Shandong University, Jinan, Shandong China

**Keywords:** Autophagy, Senescence

## Abstract

Sarcopenia, a degenerative condition marked by progressive skeletal muscle atrophy and impaired regeneration, is closely associated with aging, chronic inflammation, and disrupted proteostasis. While macroautophagy has been extensively studied in this context, little of the role of chaperone-mediated autophagy (CMA) has been known. In this study, we identified C1q/TNF-related protein 9 (CTRP9) as a novel autocrine myokine secreted by skeletal muscle that exerts dual protective functions—pro-differentiative and anti-atrophic. By using a replicative senescence model in C2C12 myoblasts, we observed that CTRP9 expression declined with cellular aging, accompanied by reduced levels of lysosome-associated membrane protein type 2A (LAMP2A), increased nucleotide-binding domain, leucine-rich-containing family, and pyrin domain-containing-3 (NLRP3) accumulation, and elevated interleukin-1β (IL-1β) secretion. Similar molecular signatures were detected in skeletal muscle tissues of CTRP9 knockout (KO) mice, further validating its role in vivo. Treatment with the biologically active globular domain of CTRP9 (gCTRP9) restored LAMP2A expression, enhanced CMA activity, and promoted selective degradation of NLRP3, thereby alleviating inflammatory stress and cellular senescence. Functionally, gCTRP9 restored myogenic differentiation markers (e.g., MYOD1) while suppressing atrophy-related genes (e.g., Fbxo32) and improving fusion potential and myotube integrity. In primary human myoblasts isolated from elderly individuals, CTRP9 and LAMP2A were significantly downregulated, and NLRP3 expression was increased—changes that were partially reversed upon gCTRP9 treatment. These findings collectively demonstrate that the CTRP9–LAMP2A–NLRP3 axis plays a pivotal role in regulating both muscle regeneration and maintenance. By targeting CMA-mediated NLRP3 degradation, CTRP9 offers a promising therapeutic strategy for combating sarcopenia through coordinated modulation of differentiation pathways and muscle atrophy.

## Introduction

Sarcopenia refers to the gradual decline in skeletal muscle mass and strength associated with normal aging [1, 2]. This loss of muscle mass and strength in older adults often leads to reduced physical performance and an increased risk of falls likely resulting in injuries such as fractures and impairments in daily living activities [3]. Several molecular mechanisms have been identified as contributors to age-related muscle wasting, including alterations in muscle fiber composition, impaired differentiation, proliferation of myoblast cells, and activation of inflammatory pathways [1, 4].

Chaperone-mediated autophagy (CMA) is another type of autophagic process that specifically targets proteins with a KFERQ amino acid sequence for degradation [5–7]. LAMP2A is identified as the rate-limiter molecule of CMA, with its level affecting the extent of CMA activity [8].

CMA is associated with various age-related pathologies, including neuronal degeneration observed in Alzheimer’s and Parkinson’s diseases [9, 10]. In aged mice, CMA activity has been shown to decline due to decreased levels of its receptor, LAMP2A, in both the liver and skeletal muscle [11, 12]. However, the role of CMA in skeletal muscle aging and sarcopenia has remained unclear in the current literature. Previous studies demonstrated the NLRP3 inflammasomes as a culprit in the manifestation of several age-related conditions, including sarcopenia [13, 14]. Recent studies have shown that CMA regulates NLRP3 inflammasome activation by facilitating the degradation of the NLRP3 protein via the CMA-lysosomal pathway [15, 16].

C1q-TNF-related protein-9 (CTRP9) is a newly identified adipocytokine that regulates glycolipid metabolism and exhibits antioxidant, anti-inflammatory and endothelial-enhancing properties. Recent findings have revealed that CTRP9 is also secreted by cardiomyocytes, where it acts as a local cardiac factor through paracrine and autocrine mechanisms [17–19]. Our previous study demonstrated that globular CTRP9 (gCTRP9) protects cardiomyocytes from palmitic acid-induced oxidative stress by enhancing autophagic flux [17]. Moreover, the deletion of C1q/TNF-related protein-9 exacerbates cardiac fibrosis in diabetic mice by regulating YAP-mediated autophagy [18]. Additionally, CTRP9 alleviates diet-induced obesity by enhancing the formation of autophagy initiation complexes, which promoted lipolysis [19].

Growing evidence suggests that CTRP9 also contributes to the mitigation of the aging process [20–22]. Furthermore, CTRP9 ameliorates both arthritis and atherosclerosis by inhibiting NLRP3 inflammasome activation [23, 24]. In our study, we demonstrated that CTRP9 is produced by skeletal muscle and C2C12 cells, functioning as an autocrine myokine.

Specifically, CTRP9 exerts a negative regulatory effect on NLRP3 protein homeostasis via the LAMP2A-mediated CMA system. A deficiency in CTRP9 results in elevated levels of NLRP3 protein and increased production of IL-1β, which impair myogenesis and lead to depletion of myofibers. Unexpectedly, we found that progressive sarcopenia is characterized by dysfunctional CMA, revealing a novel mechanism underlying age-related sarcopenia. These findings provide new perspectives for combating sarcopenia and highlighted CTRP9 as a potential therapeutic target.

## Materials and methods

### Participants and muscle sample collection

A total of 86 healthy adult participants were recruited between November 2023 and September 2024 from Qilu Hospital of Shandong University and Shunyu Community Hospital. All participants provided written informed consents before enrollment. Based on age, participants were stratified into three groups: a young group (18–34 years, 15 males and 14 females, mean age 32.86 ± 7.51 years), a middle-aged group (45–59 years, 12 males and 13 females, mean age 50.00 ± 3.42 years), and an elderly group (≥60 years, 15 males and 17 females, mean age 72.31 ± 4.96 years).

Inclusion criteria included meeting the designated age range for each group, having no significant medical history, and being willing to participate voluntarily. Exclusion criteria included: medication use within the past three months, any diagnosed clinical disorders, pregnancy or menstruation for female participants, and unwillingness to cooperate. All subjects underwent a thorough physical examination to ensure normal baseline physiological function. The demographic and clinical characteristics of participants are summarized in Supplementary Table 1.

Gastrocnemius muscle biopsy samples were obtained from patients who had undergone orthopedic surgery at Qilu Hospital and were used for the isolation of human primary myoblasts. Following informed consent, approximately 0.5 cm × 0.5 cm × 0.5 cm of muscle tissue was collected from each donor. Exclusion criteria for biopsy donors included: body mass index (BMI) < 18, muscle developmental abnormalities, bone marrow-related muscular disorders, and physical disabilities. To minimize the confounding effects of prior injury, only patients who had fully recovered from acute injuries and exhibited no signs of acute inflammation before surgery were included. Samples were grouped on the basis of donor age to ensure relatively consistent levels of physical activity across groups.

This study was approved by the Ethics Committee of Qilu Hospital of Shandong University (Approval No. KYLL-202306(XZ)-028-1), and the biopsy procedures were separately approved by the Ethics Committee of Qilu Hospital of Shandong University (Approval No. KYLL-202504-032). All procedures were conducted by following the Declaration of Helsinki and the CIOMS International Ethical Guidelines for Biomedical Research.

### Animals and protocols

The Laboratory Animal Ethics Committee of Qilu Medical Hospital, Shandong University, approved the animal experiments (Ethics Reference No. DWLL-23101). All animal procedures were conducted according to the ARRIVE guidelines.

C57BL/6 J wild-type (WT) mice were purchased from Weitong Lihua Laboratory Animal Technology Co., Ltd. (Beijing, China). CTRP9 knockout (KO) mice on a C57BL/6J background were generated and maintained by Biomodel Organism Science & Technology Development Co., Ltd. (Shanghai, China).

A power analysis was conducted by using PASS software (NCSS, USA), which indicated that a sample size of 10 mice per group would provide adequate statistical power (≥80%) to detect biologically relevant differences. A total of 40 male mice were randomly assigned to four groups (*n* = 10 per group): young WT mice (3 months), old WT mice (23 months), young KO mice (3 months), and old KO mice (23 months). Only male mice were used to avoid the confounding influence of sex hormones on skeletal muscle physiology and aging phenotypes. No animals or samples were excluded from the analysis unless pre-defined quality control criteria were not met.

Mice were housed in a specific-pathogen-free (SPF) facility under controlled environmental conditions (temperature, 22 ± 2°C; humidity, 50 ± 10%; and a 12-h light/dark cycle). Animals had ad libitum access to standard chow and water. At the end of the experimental period, a series of physiological and biochemical assays were performed as described below.

### Collection of human serums

After obtaining informed consent, participants fasted for at least 12 h before blood collection. A total of 10 mL of blood was drawn from the elbow vein into sterile, dry tubes, allowed to coagulate for 2 h at room temperature, and centrifuged at 1000 × *g* for 20 min. Fresh serum samples were either processed immediately or stored at −80 °C for later analysis.

### Tissue processing

Mice were anesthetized, euthanized, and weighed. Bilateral gastrocnemius muscles were excised, weighed, and measured for tibia length. The muscles were fixed in 4% paraformaldehyde, dehydrated with ethanol gradients, and embedded in paraffin for 7 μm sections.

### Cell culture

C2C12 myoblasts (Procell, China) were cultured in Dulbecco’s Modified Eagle Medium (DMEM) supplemented with 10% fetal bovine serum (FBS, Excellbio, China) and 1% penicillin-streptomycin (Solarbio, China). Cells were passaged based on replicative senescence criteria, with cells below 14 mean population doublings (MPD) considered young and those above 21 MPD classified as senescent [25–27]. To induce myogenic differentiation, C2C12 myoblasts were switched to differentiation medium consisting of DMEM supplemented with 2% horse serum. After 5–7 days of culture, the cells differentiated into mature multinucleated myotubes.

The C2C12 cell line was purchased from a certified vendor and was authenticated by the supplier. Routine mycoplasma testing was performed using a PCR-based detection kit, and all cultures used in this study tested negative.

Fresh gastrocnemius muscle (0.5 cm³) was minced and digested with 0.2% collagenase type II (Yeason, Cat# 40508ES60) and 0.25% trypsin at 37 °C for 2 h. The suspension was filtered, centrifuged, and resuspended in DMEM/F12 medium (Gibco, Cat# 11320033) supplemented with 20% fetal bovine serum (FBS), 1% penicillin-streptomycin, and 10 ng/mL basic fibroblast growth factor (bFGF) (PHG0368, Peprotech). Cells were cultured on Matrigel (40183, Yeason) coated dishes at 37 °C with 5% CO₂. After four rounds of differential adhesion, myogenic cells were enriched and confirmed by MyoD1 immunostaining (18943-1-AP, Proteintech). Cells at passages 2–4 were used for experiments. Myotube differentiation was induced by switching to DMEM containing 2% horse serum.

### RNA interference

Small interfering RNAs (siRNAs) targeting CTRP9 and LAMP2A (GenePharma, Shanghai, China) were transfected into C2C12 cells by using Lipo8000 (Beyotime, China).

To assess myotube atrophy, siRNA transfection was performed on C2C12 myotubes at day 4 of differentiation, and cells were harvested on day 7 for endpoint analysis [28]. To assess myogenic differentiation capacity, senescent C2C12 myoblasts were transfected with siRNA and cultured in low-serum differentiation medium (DMEM supplemented with 2% horse serum). Myogenic differentiation was evaluated between days 3 and 5 after induction, as previously described [29].

In parallel, primary human skeletal muscle cells were transfected with LAMP2A siRNA by using Lipo8000 according to the manufacturer’s protocol. After they were cultured in low-serum differentiation medium (DMEM supplemented with 2% horse serum), the cells underwent phenotypic analysis on the basis of the experimental design. The siRNA sequences targeting CTRP9 and LAMP2A, as well as the negative control, are provided in Supplementary Table 2.

### Grip force test

Muscle strength was assessed by using a Handpi HP-5N grip force tester. Each mouse underwent three tests, and the highest value was recorded.

### Hanging grid test

Mice were placed on a grid, which was then inverted 50 cm above a soft mat. Suspension impulse was calculated by multiplying the fall time by the mouse’s body weight. Three trials were conducted for each mouse with a minimum 30-min interval between trials.

### Exhaustive running tests

Mice were acclimated to treadmill running at 10 m/min for 10 min on two consecutive days. On the third day, an exhaustive running test began at 5 m/min with a 0° incline. Speed and incline were gradually increased every 5 min to a maximum of 20 m/min and 14° incline. Exhaustion was defined as the inability to remain on the track for 20 s despite the presence of external stimuli.

### Dual-energy X-ray absorption analysis of body composition

Four randomly selected mice from each group were anesthetized, weighed, and affixed to foam boards for lean mass analysis by using DEXA.

### ELISA

IL-1β and CTRP9 concentrations were measured by using ELISA kits (Proteintech, China; Boster, China) according to the manufacturers’ protocols. Absorbance at 450 nm was recorded after a 3-hour incubation at 37 °C.

### Protein blotting

Proteins were separated by SDS-PAGE and transferred onto a PVDF membrane. The membrane was blocked with 5% skimmed milk at room temperature for 1 h and then incubated overnight with primary antibodies,including anti-p21 (A22460, ABclonal, Shanghai, China; ab188224, Abcam, Cambridge, UK), anti-Tubulin (66031-1-lg, Proteintech,Wuhan, China), anti-CTRP9 (bs-15085R, Bioss, Beijing, China), anti-GAPDH (66004-1-lg, Proteintech, Wuhan, China), anti-p53 (ab26, Abcam, Cambridge, UK), anti-GLB1/β-galactosidase (15518-1-AP, Proteintech, Wuhan, China), anti-Caspase-1/P20 (22915-1-AP, Proteintech, Wuhan, China), anti-Hsc70 (10654-1-AP, Proteintech, Wuhan, China), anti-Fbxo32 (ab168372, Abcam, Cambridge, UK), anti-NLRP3 (19771-1-AP and 68102-1-Ig, Proteintech, Wuhan, China), anti-LAMP2A (ab125068, Abcam, Cambridge, UK; ET1601-24, Huaan Biotechnology, Hangzhou, China), anti-ASC (10500-1-AP, Proteintech, Wuhan, China), anti-IL-1β (12242, Cell Signaling Technology, Danvers, MA, USA), anti-LC3B (ab192890, Abcam, Cambridge, UK), anti-IL-18 (10663-1-AP, Proteintech, Wuhan, China), anti-p16INK4A (10883-1-AP，Proteintech，Wuhan，China；PT0242R, Immunoway, Suzhou, CHINA), anti-p19ARF (83259, Cell Signaling Technology, Danvers, MA, USA), and anti-MyoD1 (18943-1-AP, Proteintech, Wuhan, China). After being washed, the membranes were incubated with horseradish peroxidase-conjugated secondary antibodies (ZB-2305 or ZB-2301, ZSGB-BIO, Beijing, China).

### Real-time quantitative PCR (RT-qPCR)

Total RNA was extracted by using the RNA Fast200 kit (Fastagen, Hangzhou, China), and reverse transcription quantitative PCR (RT-qPCR) was performed by using the SYBR Premix Ex Taq kit (Yeason, Shanghai, China). GAPDH was used as an internal control. The primers for specific genes are listed in Supplementary Table S3.

### Senescence-associated-galactosidase activity determination

Cells were washed with phosphate-buffered saline (PBS), fixed with the manufacturer’s fixative, and stained with Senescence-associated actosidase (SA-β-gal) staining solution (Beyotime C0602, Shanghai, China). Images were captured by using a Nikon TiS microscope (Japan).

### Immunofluorescence staining and image analysis

After fixation and blocking, cells were incubated overnight at 4 °C with the following primary antibodies: anti-myosin heavy chain (MHC, Cat# 376157, Santa Cruz Biotechnology, Dallas, TX, USA), anti-NLRP3 (Cat# 68102, Proteintech, Wuhan, China), anti-LAMP2A (Cat# ab125068, Abcam, Cambridge, UK), and anti-MyoD1 (Cat# 18943, Proteintech, Wuhan, China). The next day, cells were incubated with Alexa Fluor 488– or Alexa Fluor 594–conjugated secondary antibodies (Abcam, Cambridge, UK) for 1 h at room temperature. Nuclei were stained with DAPI (Beyotime, Haimen, China) for 5 min, followed by imaging.

Confocal images were captured by using a laser scanning confocal microscope (LSM 900, Carl Zeiss, Oberkochen, Germany). Immunofluorescence images of MYH were acquired by using the ImageXpress® Micro Confocal High-Content Imaging System (Molecular Devices, USA), with five random fields captured per well.

For myotube analysis, the length (along the longitudinal axis) and width (perpendicular to the axis) of individual myotubes were measured by using ImageJ software. At least 100 myotubes per group were analyzed for statistical evaluation. For C2C12 differentiation assessment, the fusion index was calculated as the number of nuclei within MHC-positive multinucleated myotubes (≥2 nuclei per myotube) divided by the total number of DAPI-stained nuclei.

### HE staining and muscle fiber analysis

Gastrocnemius muscles were fixed in 4% paraformaldehyde, dehydrated, paraffin-embedded, and sectioned (8 μm). Deparaffinized sections were stained with hematoxylin (5 min) and eosin (1 min). Images were captured using the Olympus VS200 slide scanner (Olympus Corporation, Tokyo, Japan) and analyzed with Image Pro Plus. The cross-sectional area (CSA) was measured by analyzing 100 intact fibers from 5 randomly selected fields per mouse by using ImageJ. Data were expressed as mean ± SEM.

### Immunohistochemical staining

After deparaffinization and hydration, the supporting information provides detailed procedures and antibodies. Images were captured by using a Pannoramic Scan (3D Histech, Budapest, Hungary) and analyzed with Image Pro Plus. The cross-sectional area of fast-twitch muscle fibers were calculated by using the same method as described above, and the cross-sectional areas of all slow-twitch muscle fibers were measured and averaged. The ratio of slow to fast muscle fibers was determined by the count of slow MHC-positive fibers divided by the count of fast MHC-positive fibers.

### In situ muscle contraction performance

Mice were anesthetized and positioned on a temperature-controlled plate. The gastrocnemius muscle was exposed, detached proximally, and connected distally to a force transducer via surgical suture. An isolated pulse stimulator delivered a 5-ms, 5-V square-wave pulse to determine the optimal initial muscle length. The muscle was then stimulated with a 25-ms, 5-V pulse to record the maximum tetanic force. Saline was applied continuously to maintain physiological conditions. After the experiment, the gastrocnemius muscle was excised, and the mice were euthanized.

### Blinding procedures

To minimize potential bias during data acquisition and analysis, several assessments were performed in a blinded manner. Specifically, immunofluorescence images used for myotube morphological measurements (e.g. diameter, length) and fusion index quantification were randomized and analyzed by investigators blinded to group assignments. SA-β-gal staining images and cross-sectional area (CSA) measurements of muscle fibers were also assessed under blinded conditions to ensure objectivity in evaluating senescence-associated phenotypes. For Western blot and qPCR analyses, blinding was not applied, as data were quantified by using automated software based on band intensity or Ct values.

### Statistical analysis

Data were analyzed by using SPSS and GraphPad Prism 10. Before applying parametric tests, the normality of the data distribution was assessed by using the Shapiro–Wilk test. Homogeneity of variances was tested by using Levene’s test. Differences between the two groups were evaluated with unpaired *t*-tests, while one-way or two-way ANOVA, followed by Tukey’s post hoc test, was used for comparisons involving three or more groups. Statistical significance for Pearson’s correlation analysis was determined by using a *p*-value threshold of 0.05.

## Results

### Serum and muscle CTRP9 levels decrease in the elderly, and CTRP9 KO worsens sarcopenia in aging mice

**Fig. 1:**
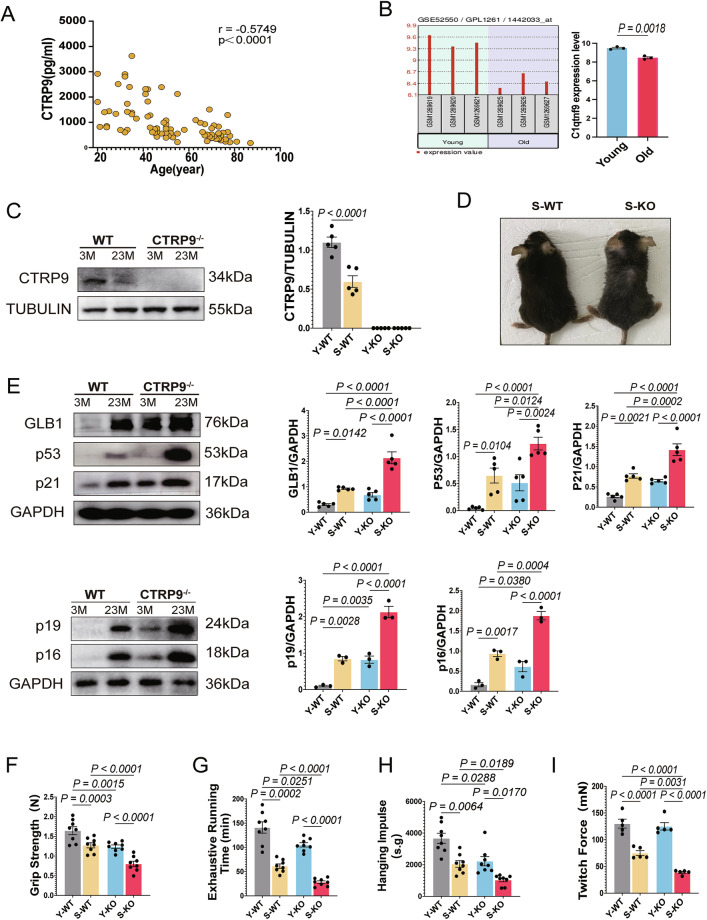
CTRP9 expression is reduced in elderly individuals, and CTRP9 deficiency exacerbates sarcopenia in aging mice. **A** Pearson correlation analysis of serum CTRP9 levels in 87 human participants spanning different age groups. **B** CTRP9 mRNA expression in gastrocnemius muscle from young and aged healthy male C57BL/6J mice (*n* = 3 per group) analyzed by using the GSE52550 dataset. **C** Western blot analysis of CTRP9 expression in gastrocnemius muscle from 3-month-old and 23-month-old WT and CTRP9 KO mice; TUBULIN serves as the loading control (*n* = 5). **D** Representative photographs of 23-month-old WT and CTRP9KO mice. **E** Western blot analysis of GLB1, p53, p21, p19 and p16 expression in gastrocnemius muscle from 3-month-old and 23-month-old WT and CTRP9KO mice; GAPDH serves as the loading control (*n* = 5 for GLB1, p53 and p21; *n* = 3 for p16 and p19). **F** Grip strength measurements (N) in WT and CTRP9KO mice across different age groups (*n* = 8). **G** Exhaustive running time (min) in WT and CTRP9KO mice across different age groups (*n* = 8). **H** Hanging grid test performance (s.g) in WT and CTRP9KO mice across different age groups (*n* = 8). **I** Quantitative analysis of individual twitch force (mN) in the gastrocnemius muscle from WT and CTRP9KO mice across different age groups (*n* = 5). Data are presented as mean ± SEM.

To assess the relationship between CTRP9 and aging, serum levels were measured in 86 healthy individuals, revealing a significant inverse correlation with age (Fig. 1A, Supplementary Table S1). Transcriptomic data from GSE52550 and Western blot analysis confirmed decreased CTRP9 expression in aged mouse muscle (Fig. 1B, C).

To elucidate the role of CTRP9 in sarcopenia, WT and CTRP9 KO mice were studied at 3 and 23 months of age. In 23-month-old CTRP9 knockout (KO) mice, signs of sarcopenia—such as reduced body weight and dull coat—were more pronounced than in aged wild-type (WT) mice (Fig. 1D, Supplementary Fig. S1A). Age-related markers (GLB1, p53, p21, p16, p19) were elevated in the gastrocnemius muscle of aged mice, with a further increase observed in CTRP9 KO mice compared with aged WT controls (Fig. 1E).

**Fig. 2:**
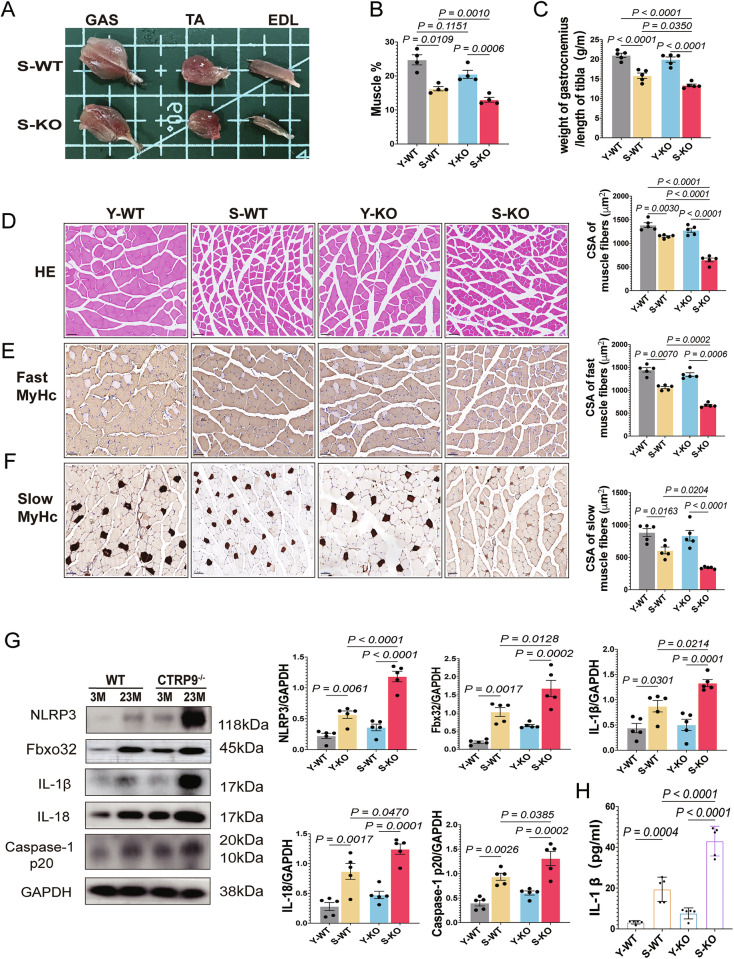
CTRP9 KO exacerbates muscle fiber atrophy and inflammation in aging mice. **A** Representative images of gastrocnemius (GAS), tibialis anterior (TA), and extensor digitorum longus (EDL) muscles from different groups. **B** Lean body mass percentage determined by dual-energy X-ray absorptiometry (DEXA) (*n* = 4). **C** Ratio of gastrocnemius weight to tibia length (g/m) in WT and CTRP9KO mice across different age groups (*n* = 5). Data are presented as mean ± SEM. **D** Hematoxylin-eosin (HE) staining of muscle fibers and analysis of cross-sectional area (CSA, μm²) (*n* = 5). Scale bar = 50 μm. **E** Immunostaining of fast-twitch muscle fibers and corresponding CSA (μm²) (*n* = 5). Scale bar = 50 μm. **F** Immunostaining of slow-twitch muscle fibers and corresponding CSA (μm²) (*n* = 5). Scale bar = 50 μm. **G** Western blot analysis of NLRP3, Fbxo32, IL-1β, IL-18 and Caspase-1 P20 expression in gastrocnemius muscle from 3-month-old and 23-month-old WT and CTRP9 KO mice; GAPDH serves as the loading control (*n* = 5). **H** Quantification of IL-1β secretion in gastrocnemius muscle using ELISA (*n* = 5). Data are presented as mean ± SEM.

Sarcopenic phenotypes were evaluated via muscle strength and mass. CTRP9 KO aged mice exhibited greater declines in grip strength, running endurance, hanging time, and tetanic force (Fig. 1F–I). DEXA analysis and muscle-to-tibia length ratios confirmed aggravated muscle mass loss in CTRP9-deficient aged mice (Fig. 2A–C). These findings indicate that CTRP9 deficiency exacerbates age-associated molecular features of sarcopenia.

### CTRP9 deficiency enhances muscle atrophy and inflammation

Histology showed reduced fiber size in aged WT mice, which was worsened in CTRP9 KO mice (Fig. 2D–F). Fast and slow-twitch fiber cross-sectional area (CSA) decreased significantly, especially in KO mice. Western blot revealed increased Fbxo32 expression in aged WT and KO mice, indicating enhanced muscle atrophy (Fig. 2G). These findings suggest that CTRP9 deficiency aggravates age-related muscle atrophy, supporting a protective role for CTRP9 in the context of sarcopenia.

To further investigate the mechanisms underlying CTRP9-mediated regulation of sarcopenia, we assessed the activation of the NLRP3 inflammasome, a key inflammatory complex composed of NLRP3, ASC, and pro-Caspase-1. Previous studies have shown that inflammasome activity increases with aging, characterized by elevated levels of NLRP3 and pro-Caspase-1 and their cleavage into active forms [30]. Consistent with this, in our study, Western blot analysis demonstrated significantly increased NLRP3, IL-18 and IL-1β protein levels in aged WT mice relative to young WT mice, with further elevation in aged CTRP9 KO mice (Fig. 2G).

Inflammasome activation promotes the cleavage of pro-Caspase-1 into active Caspase-1, which mediates the maturation of IL-18 and IL-1β [30, 31]. Accordingly, in our study, levels of mature IL-1β were significantly elevated in both aged WT and KO mice, indicating enhanced Caspase-1 activation and subsequent processing of pro-IL-1β (Fig. 2H). In short, these results demonstrate that CTRP9 deficiency intensifies NLRP3 inflammasome activation, thereby promoting inflammation and contributing to sarcopenia. This means that it underscores the critical role of CTRP9 in maintaining skeletal muscle homeostasis and preventing age-related degeneration.

### Replicative senescence model is successfully established in C2C12 cells, accompanied by reduced CTRP9 expression

To investigate the role of CTRP9 in skeletal muscle aging, we established a replicative senescence model by using high-passage C2C12 myoblasts (*P* > 35), as previously described [32, 33]. These cells exhibited typical senescence features, including G0/G1 cell cycle arrest, increased SA-β-galactosidase activity, elevated p16, p21, and p53 expression, as well as impaired myogenic differentiation characterized by shorter and thinner myotubes. SA-β-gal and Giemsa staining confirmed increased senescence and reduced differentiation capacity in senescent cells compared with early-passage controls (Supplementary Fig. S2A). Western blot and qPCR analyses further demonstrated upregulation of senescence markers (p53, p21) and the DNA damage marker H2AX (Supplementary Fig. S2B, C), validating the successful establishment of the model. Notably, CTRP9 protein levels were significantly reduced in senescent cells (Supplementary Fig. S2D), suggesting a potential link between CTRP9 downregulation and cellular aging.

To evaluate the therapeutic potential of CTRP9, senescent myoblasts were treated with 5 μg/mL of recombinant globular CTRP9 (gCTRP9), its biologically active isoform [20]. Western blot analysis revealed a time-dependent reduction in p53 and p21 expression over 0–36 h (Supplementary Fig. S2E), indicating that gCTRP9 supplementation may alleviate cellular senescence in aging myoblasts.

### CTRP9 knockdown promotes senescence and atrophy in differentiated myotubes

**Fig. 3:**
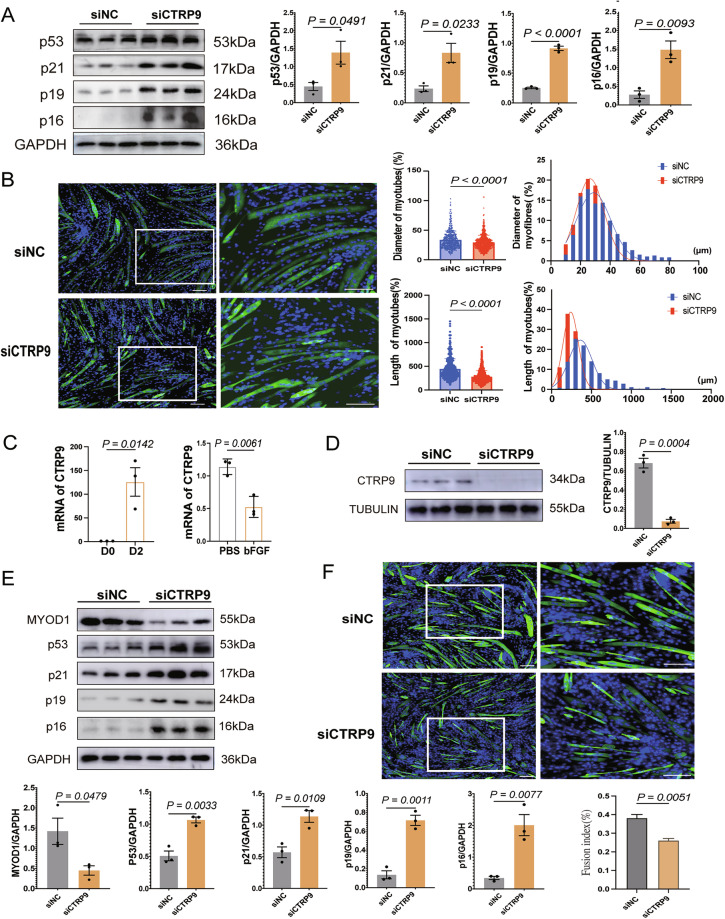
CTRP9 knockdown accelerates senescence and atrophy in differentiated C2C12 myotubes and impairs myogenesis in replicative aging C2C12 myoblasts. **A** Western blot analysis of p53, p21, p16 and p19 protein expression in C2C12 myotubes transfected with siCTRP9 or siNC; GAPDH serves as the loading control (*n* = 3). **B** Immunofluorescence staining of MHC (green) and DAPI (blue) in C2C12 myotubes transfected with siCTRP9 or siNC. The diameter and length of myotubes are measured, and their nuclei are counterstained with DAPI. Representative images are shown (*n* = 3). Scale bar = 100 μm. **C** Left: CTRP9 mRNA expression in C2C12 cells at day 0 and day 2 of differentiation. Right: CTRP9 mRNA expression in C2C12 cells treated with PBS or bFGF (10 ng/mL) during differentiation. CTRP9 expression is assessed by qPCR (*n* = 3). **D** Western blot analysis of CTRP9 protein expression in siNC and siCTRP9 groups; TUBULIN serves as the loading control (*n* = 3). **E** Western blot analysis of MYOD1, p53, p21, p19 and p16 protein expression in siNC and siCTRP9 groups; GAPDH serves as the loading control (*n* = 3). **F** Immunofluorescence staining of myosin heavy chain (MHC; green) in the siNC and siCTRP9 groups of C2C12 myoblasts. Nuclei were counterstained with DAPI (blue) (*n* = 3). Scale bar = 100 μm. Data are presented as mean ± SEM.

To investigate the role of CTRP9 in differentiated myotubes, replicative senescent C2C12 cells were transfected with CTRP9-specific siRNA on day 4 of differentiation, and analyzed on day 7. qPCR confirmed effective CTRP9 knockdown in the siCTRP9 group (Supplementary Fig. S3A). SA-β-gal staining revealed a marked increase in positive staining area following CTRP9 silencing (Supplementary Fig. S3B). Western blot analysis further showed upregulation of p53 and p21, indicating activation of the p53-dependent senescence pathway, along with elevated p16 and p19 levels, suggesting upregulation of INK4a/ARF locos, which may coordinately regulate both Rb and p53 pathways (Fig. 3A) [34]. Additionally, MHC immunofluorescence staining demonstrated significantly reduced myotube length and diameter in the siCTRP9 group, indicating exacerbated myotube atrophy (Fig. 3B). Together, these findings suggest that CTRP9 knockdown aggravates the progression of cellular senescence and significantly promotes myotube atrophy in differentiated C2C12 cells.

### CTRP9 deficiency accelerates senescence in replicative aging C2C12 myoblasts and compromises their myogenic differentiation potential

To explore the role of CTRP9 during early differentiation, we first examined its expression dynamics during myogenesis. qPCR analysis revealed that CTRP9 mRNA levels significantly increased on day 2 of differentiation compared with day 0 (Fig. 3C, left). This upregulation was suppressed by bFGF treatment (10 ng/mL), which inhibits myogenic differentiation, suggesting that CTRP9 expression is closely linked to myogenic progression and is downregulated when differentiation is inhibited. (Fig. 3C, right).

To assess its functional role, replicative senescent C2C12 myoblasts were transfected with CTRP9-specific siRNA one day prior to differentiation induction (Fig. 3D). SA-β-gal staining revealed increased senescence in the siCTRP9 group (Supplementary Fig. S3C), while Western blot analysis showed elevated expression of p53, p21, p16, and p19 (Fig. 3E), indicating activation of both the p53 and Rb senescence pathways.

Given that myogenic differentiation relies on key regulatory factors such as MyoD1 and myosin heavy chain (MHC) [35], we examined the effect of CTRP9 knockdown on myotube formation. Previous studies have shown that the expression of these markers is significantly impaired in senescent myoblasts [32, 36]. MHC immunofluorescence on day 5 revealed reduced myotube formation and fusion index in the siCTRP9 group (Fig. 3F) suggesting impaired differentiation.

In brief, these findings demonstrate that CTRP9 is essential for maintaining the myogenic potential of aging myoblasts. Its deficiency exacerbates cellular senescence and compromises myogenic differentiation.

### gCTRP9 promotes NLRP3 degradation in a lysosome-dependent manner

**Fig. 4:**
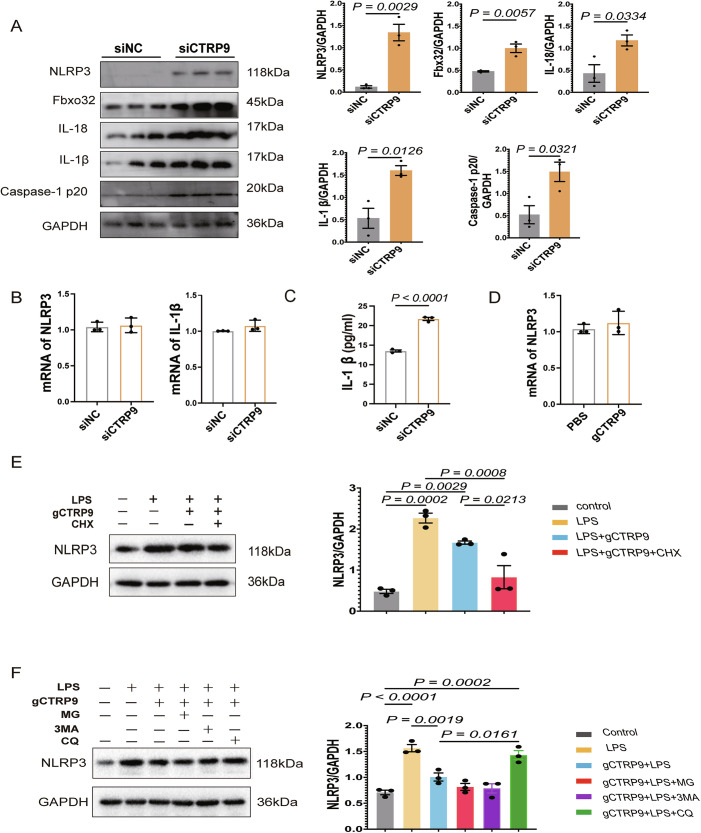
gCTRP9 promotes NLRP3 degradation in a lysosome-dependent manner. **A** Western blot analysis of NLRP3, Fbxo32, IL-18, IL-1β and Caspase-1 p20 protein expression in the siNC and siCTRP9 groups of C2C12 myotubes; GAPDH serves as the loading control (*n* = 3). **B** qPCR analysis of mRNA levels of NLRP3 and IL-1β in the siNC and siCTRP9 groups of C2C12 myotubes. (*n* = 3). **C** ELISA quantification of secreted IL-1β levels in the siNC and siCTRP9 groups of C2C12 myotubes. (*n* = 3). **D** qPCR analysis of NLRP3 mRNA levels in C2C12 myotubes treated with PBS or gCTRP9 (*n* = 3). **E** Western blot analysis of NLRP3 protein expression in C2C12 cells treated with cycloheximide (CHX) in the presence or absence of gCTRP9; GAPDH serves as the loading control (*n* = 3). **F** Western blot analysis of NLRP3 protein expression in C2C12 cells pre-treated with LPS and gCTRP9, followed by the indicated inhibitors: MG132 (proteasome inhibitor), chloroquine (CQ, lysosomal inhibitor), and 3-methyladenine (3-MA, autophagy inhibitor); GAPDH serves as the loading control (*n* = 3). Data are presented as mean ± SEM.

Previous studies suggested that NLRP3 inflammasome activation inhibited myotube growth without affecting protein degradation pathways [37]. To investigate whether CTRP9 modulates NLRP3 inflammasome activity, we silenced CTRP9 in senescent C2C12 myotubes by using siRNA. Immunoblotting revealed increased protein levels of NLRP3, IL-1β, IL-18, caspase-1, and the atrophy marker Fbxo32 in the siCTRP9 group, indicating that CTRP9 deficiency promotes myotube atrophy via inflammasome activation (Fig. 4A). Notably, qPCR analysis showed no change in NLRP3 or IL-1β mRNA levels (Fig. 4B), suggesting post-transcriptional regulation. Elevated IL-1β secretion in the supernatant of CTRP9-deficient cells further confirms enhanced inflammasome activity (Fig. 4C).

To determine whether CTRP9 regulates NLRP3 protein stability, C2C12 cells were treated with siCTRP9 or recombinant gCTRP9 (5 μg, 36 h). No significant changes in NLRP3 mRNA levels were observed (Fig. 4D). However, in the presence of cycloheximide (CHX), gCTRP9 markedly reduced NLRP3 protein levels, supporting the hypothesis that CTRP9 modulates NLRP3 stability (Fig. 4E).

To further make clear the mechanism of NLRP3 degradation, we explored the two major protein degradation pathways: the proteasome-dependent and lysosome-dependent pathways. We treated C2C12 cells with gCTRP9 (5 μg for 24 h), followed by lipopolysaccharide (LPS) to activate inflammasomes. Cells were subsequently treated with the proteasome inhibitor MG132 (20 μM), lysosomal inhibitor chloroquine (CQ) (50 μM), or phosphatidylinositol 3-kinase inhibitor 3-methyladenine (3-MA) (5 mM) for 6 h before harvest. Our updated experiments showed that CQ significantly blocked NLRP3 degradation induced by gCTRP9, whereas MG132 and 3-MA did not show appreciable effects. These findings suggest that CTRP9 promotes NLRP3 degradation in a lysosome-dependent manner (Fig. 4F).

### CTRP9 gene knockout reduces CMA activity by downregulating LAMP2A expression during both myoblast differentiation and in mature C2C12 myotubes

**Fig. 5:**
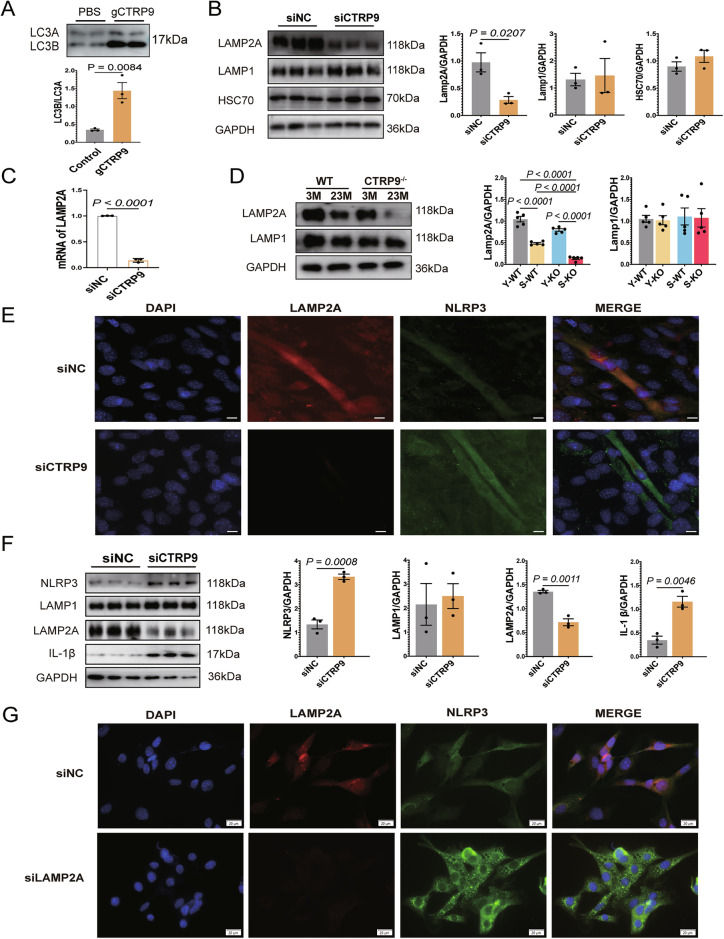
CTRP9 gene knockout reduces CMA activity by downregulating LAMP2A expression during both myoblast differentiation and in mature C2C12 myotubes. **A** Western blot analysis of LC3A and LC3B expression in siNC and siCTRP9 groups (*n* = 3). **B** Western blot analysis of LAMP2A, LAMP-1, and HSC70 expression in siNC and siCTRP9 groups; GAPDH serves as the loading control (*n* = 3). **C** The mRNA expression levels of LAMP2A in siNC and siCTRP9 groups. (*n* = 3). **D** Western blot analysis of NLRP3, pro-caspase-1, IL-1β, LAMP2A, and LAMP-1 expression in gastrocnemius muscle from 3-month-old and 23-month-old WT and CTRP9 KO mice; GAPDH serves as the loading control (*n* = 5). **E** Immunofluorescence staining of siNC and siCTRP9 C2C12 myotubes for NLRP3 (green), LAMP2A (red), and DAPI-stained nuclei (blue) (*n* = 3). Scale bar = 20 μm. **F** Western blot analysis of NLRP3, LAMP-1, LAMP2A and IL-1β expression in siNC and siCTRP9 C2C12 myoblasts; GAPDH serves as the loading control (*n* = 3). **G** Immunofluorescence staining of siNC and siCTRP9 C2C12 myoblasts for NLRP3 (green), LAMP2A (red), and DAPI-stained nuclei (blue) (*n* = 3). Scale bar = 20 μm. Data are expressed as mean ± SEM.

Building on our findings that CTRP9 promotes lysosome-dependent degradation of NLRP3, we next explored whether this regulation involves chaperone-mediated autophagy (CMA). Although gCTRP9 enhanced LC3B degradation (Fig. 5A), only CQ—but not 3-MA or MG132—blocked NLRP3 degradation, suggesting that CTRP9 acts through a mechanism independent of macroautophagy and proteasomal pathways.

Given prior reports that NLRP3 can be degraded via CMA in a LAMP2A-dependent manner [16, 38], we examined CMA components in CTRP9-deficient cells. Western blot analysis revealed significantly reduced LAMP2A protein levels in CTRP9-silenced C2C12 myotubes, while levels of LAMP-1 and HSC70 remained unchanged (Fig. 5B). Notably, LAMP2A mRNA expression remained unchanged (Fig. 5C), suggesting post-translational regulation. These findings were corroborated in vivo, where CTRP9 knockout mice exhibited a specific downregulation of LAMP2A in skeletal muscle without affecting LAMP-1 (Fig. 5D).

Immunofluorescence staining further confirms these changes: CTRP9 silencing led to a marked reduction in LAMP2A-positive vesicles and a concomitant increase in NLRP3-positive vesicles in senescent myotubes (Fig. 5E), as well as in replicative senescent myoblasts (Fig. 5F). Fluorescence imaging demonstrated reduced intensity and number of LAMP2A-positive compartments and increased NLRP3 accumulation in CTRP9-deficient cells (Fig. 5G).

Together, these findings demonstrate that CTRP9 regulates CMA activity primarily through modulation of LAMP2A expression, thereby promoting NLRP3 degradation in both differentiating and mature skeletal muscle cells.

### gCTRP9 attenuates senescence and atrophy in C2C12 myotubes by inducing LAMP2A-mediated NLRP3 degradation

To determine whether LAMP2A is required for CTRP9-mediated regulation of NLRP3, we silenced LAMP2A in senescent C2C12 myotubes by using siRNA. LAMP2A knockdown significantly increased the expression of senescence markers (p53, p21, p16, p19) and the atrophy marker Fbxo32, suggesting exacerbated cellular aging and muscle atrophy.

**Fig. 6:**
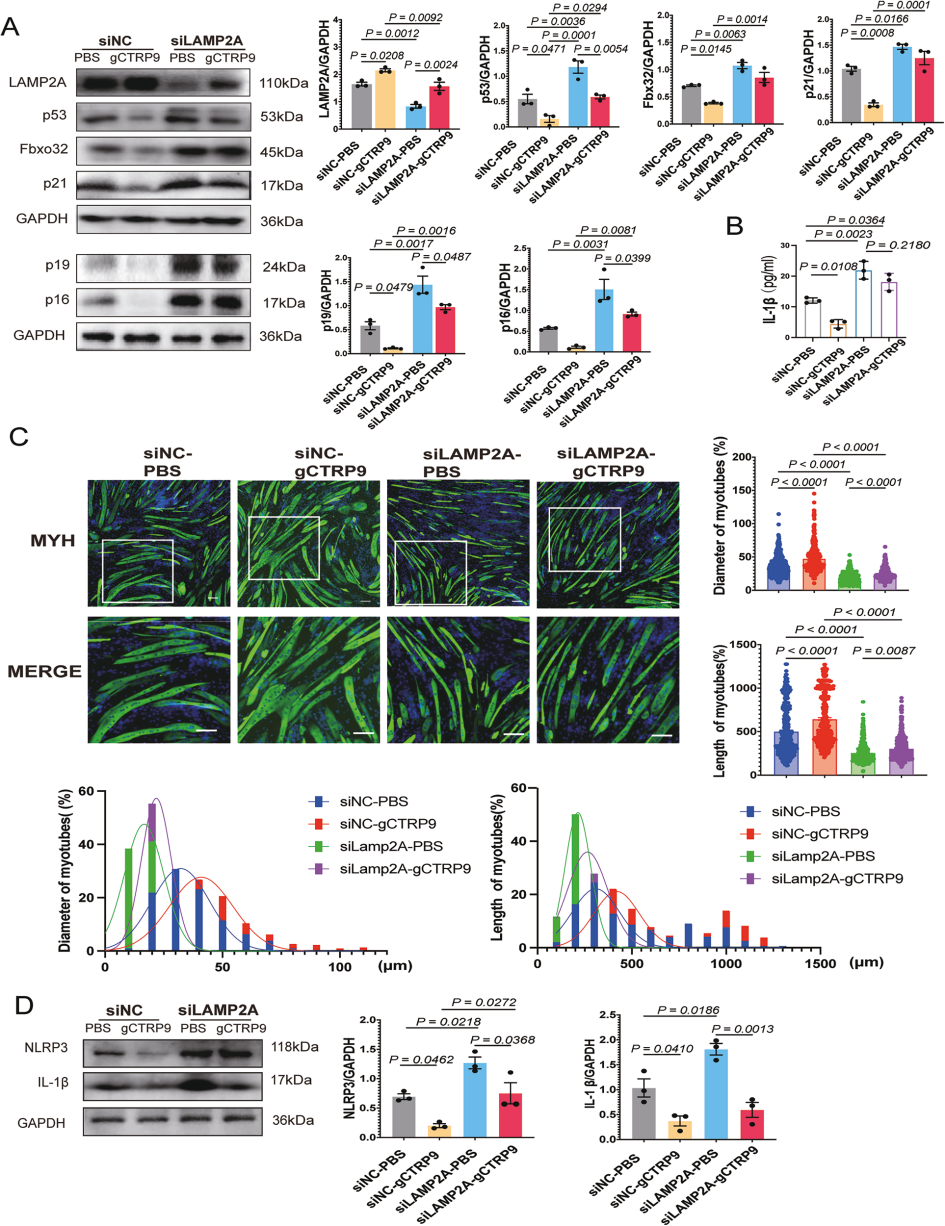
gCTRP9 mitigates senescence and atrophy in C2C12 myotubes by inducing LAMP2A-mediated NLRP3 degradation. **A** Western blot analysis of LAMP2A, p53, Fbxo32, p21, p19 and p16 expression in C2C12 myotubes; GAPDH serves as the loading control (*n* = 3). **B** ELISA quantification shows IL-1β levels in the supernatant of C2C12 myotubes (*n* = 3). **C** Immunofluorescence staining of MHC (green) and nuclei (DAPI; blue) in C2C12 myotubes treated with gCTRP9. Myotube diameter and length were measured; representative images are shown (*n* = 3). Scale bar = 100 μm. **D** Western blot analysis of NLRP3 and IL-1β expression in C2C12 myotubes; GAPDH serves as the loading control (*n* = 3). Data are expressed as mean ± SEM.

Treatment with recombinant gCTRP9 (5 μg/mL, 36 h) markedly reduced these markers at the protein level, but this protective effect was significantly diminished upon LAMP2A knockdown (Fig. 6A). Similarly, gCTRP9 reduced IL-1β secretion as measured by ELISA, an effect attenuated by LAMP2A silencing (Fig. 6B).

Immunofluorescence staining showed that gCTRP9 rescued myotube length and diameter in control cells, whereas its therapeutic effects were markedly attenuated in LAMP2A-deficient cells (Fig. 6C). In parallel, gCTRP9 suppressed NLRP3 and IL-1β expression, whereas LAMP2A knockdown partially reversed this suppression (Fig. 6D).

In short, these results indicate that gCTRP9 mitigated senescence and atrophy in C2C12 myotubes by enhancing LAMP2A-mediated chaperone-mediated autophagy (CMA) and this mechanism promoted the degradation of NLRP3, alleviated inflammation, and preserved myotube integrity.

### gCTRP9 alleviates myoblast senescence and enhances C2C12 myogenesis by promoting LAMP2A-mediated NLRP3 degradation

**Fig. 7:**
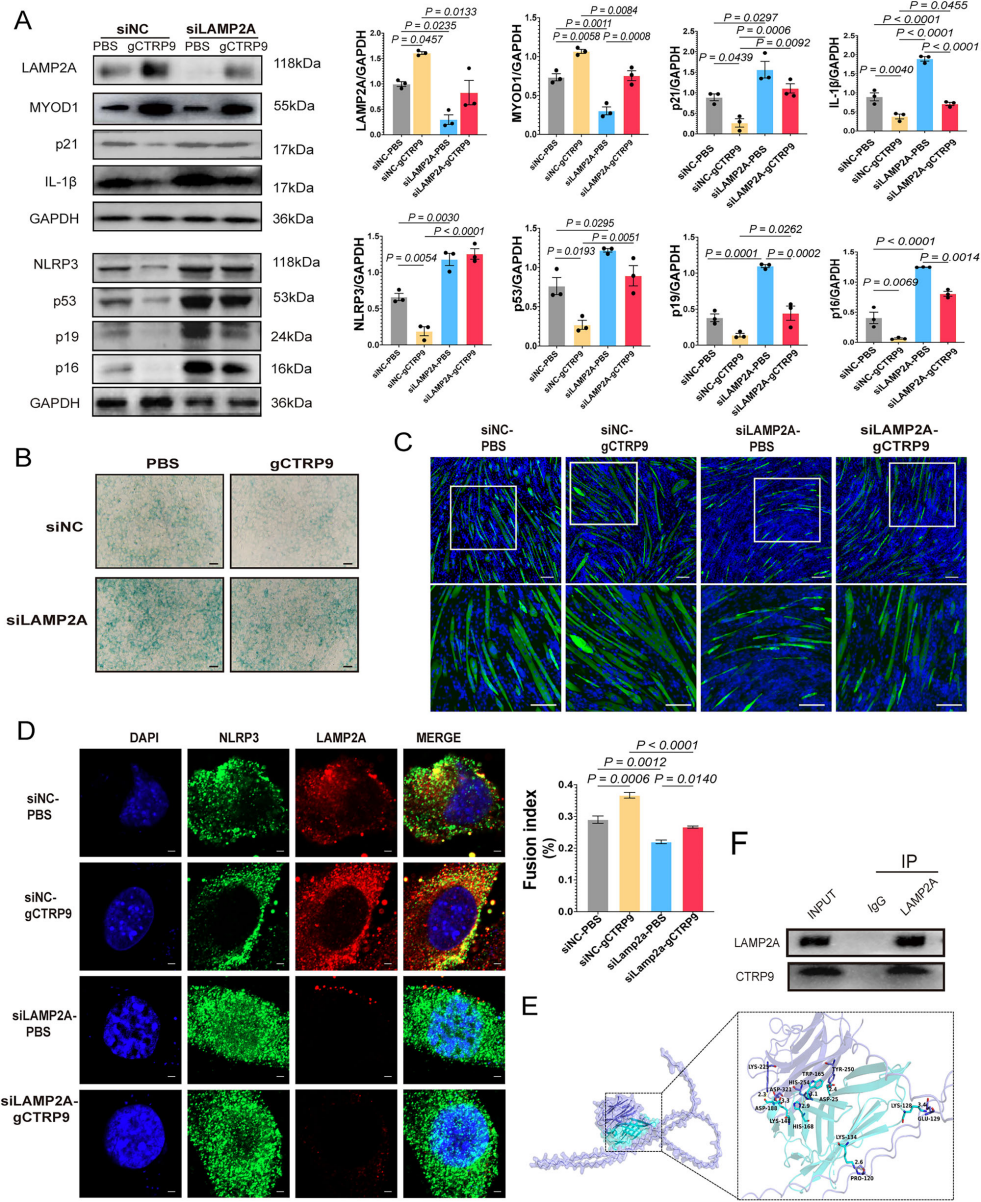
CTRP9 is essential for the induction of LAMP2A-mediated NLRP3 degradation in C2C12 myoblasts. **A** Western blot analysis of LAMP2A, MYOD1, p21, IL-1β, p53, p19 and p16 expression in the siNC and siCTRP9 groups of C2C12 myoblasts; GAPDH serves as the loading control (*n* = 3). **B** SA-β-gal staining in the siNC and siCTRP9 groups of C2C12 myoblasts (*n* = 3). **C** Immunofluorescence staining of MHC (green) in siNC and siCTRP9 C2C12 myoblasts; nuclei were counterstained with DAPI (blue). Scale bar = 100 μm (*n* = 3). **D** Confocal microscopy showing cellular co-localization of LAMP2A (red) and NLRP3 (green) in siNC and siCTRP9 groups of C2C12 myoblasts. Nuclei are counterstained with DAPI (blue). Scale bar = 20 μm. **E** Protein–protein docking model of CTRP9 (purple) and LAMP2A (blue), with interfacial residues represented as surface maps. Hydrogen-bond interactions are highlighted, and binding sites are shown as stick structures in corresponding colors. **F** Co-immunoprecipitation analysis of the interaction between CTRP9 and LAMP2A in C2C12 myotubes. Data are expressed as mean ± SEM.

To assess the role of LAMP2A in gCTRP9-mediated NLRP3 degradation and its impact on myogenesis, we downregulated LAMP2A in replicative senescent C2C12 myoblasts by using siRNA. LAMP2A knockdown significantly increased the expression of senescence markers (p53, p21, p16, p19) and inflammasome-associated proteins (NLRP3, IL-1β), indicating enhanced cellular senescence and inflammation (Fig. 7A). In contrast, gCTRP9 treatment reduced p53, NLRP3, and IL-1β levels while upregulating MYOD1, a key myogenic factor—effects largely abrogated by LAMP2A silencing.

SA-β-gal staining supported these findings, revealing an increased burden of senescent cells following LAMP2A knockdown, while the senescence-attenuating effect of gCTRP9 was markedly diminished in the absence of LAMP2A (Fig. 7B). Functionally, LAMP2A deficiency impaired myoblast differentiation and fusion, whereas gCTRP9 effectively restored these processes in control cells but failed to fully rescue them in LAMP2A-deficient myoblasts (Fig. 7C).

Confocal imaging revealed enhanced co-localization of LAMP2A and NLRP3 upon gCTRP9 treatment, an effect diminished by LAMP2A knockdown (Fig. 7D).

Together, these results indicate that gCTRP9 alleviates myoblast senescence and promotes myogenesis by facilitating LAMP2A-mediated CMA-dependent degradation of NLRP3, highlighting its therapeutic potential in mitigating skeletal muscle aging.

### CTRP9 directly interacts with LAMP2A to modulate CMA activity

To elucidate the molecular mechanism by which CTRP9 modulates LAMP2A-mediated CMA, we performed rigid protein–protein docking analysis and co-immunoprecipitation (Co-IP) assays to evaluate the interaction between CTRP9 and LAMP2A.

Rigid protein–protein docking revealed stable hydrogen bond interactions between CTRP9 and LAMP2A at specific amino acid residues, including LYS225-ASP188 and HIS254-HIS168, thus forming a stable protein docking model (Fig. 7E). These computational findings provide structural evidence of direct binding between the two proteins.

To validate this interaction in a biological context, Co-IP assays were conducted in C2C12 myotubes. The results confirm the direct interaction between CTRP9 and LAMP2A under physiological conditions (Fig. 7F).

Given that LAMP2A is the rate-limiting protein in chaperone-mediated autophagy (CMA), these findings suggest that CTRP9 is directly bound to LAMP2A at the protein level, thereby modulating CMA-mediated NLRP3 degradation. This mechanism not only alleviates myoblast senescence and promoted myogenesis but also means therapeutic potential. In this sense, gCTRP9 may serve as a promising therapeutic factor for mitigating the progression of sarcopenia.

### CTRP9-LAMP2A-NLRP3 axis is dysregulated in human primary myoblasts during aging and gCTRP9 treatment ameliorates cellular senescence

**Fig. 8:**
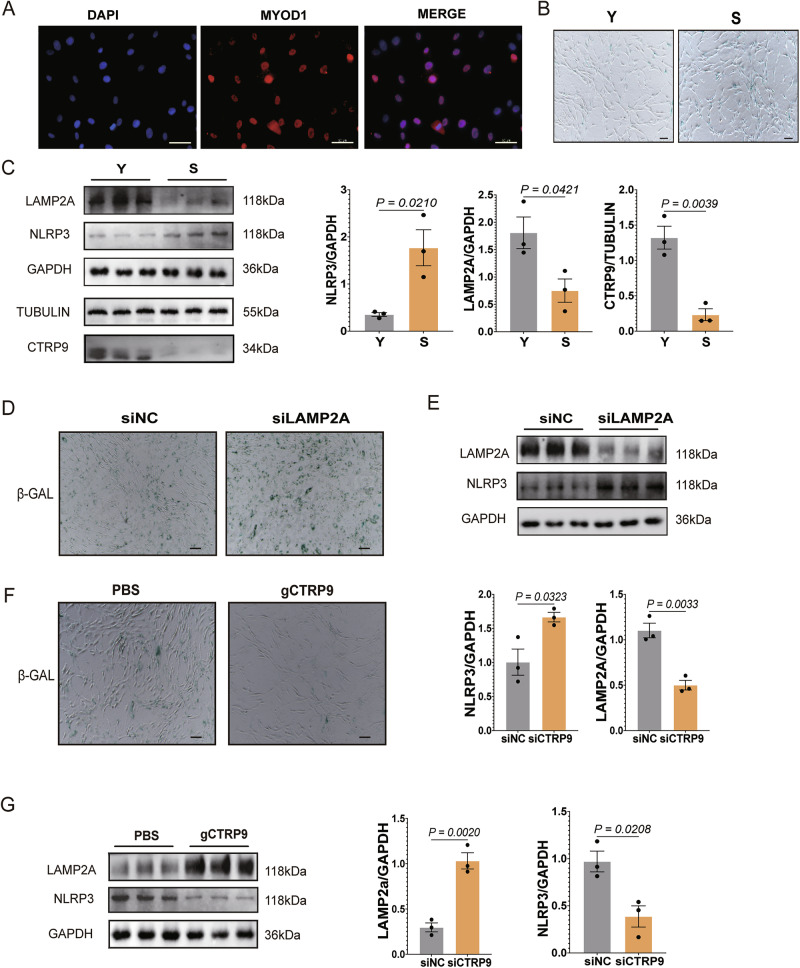
CTRP9–LAMP2A–NLRP3 axis is dysregulated in aged human primary myoblasts and restored by gCTRP9 treatment. **A** Representative immunofluorescence images of human primary myoblasts stained for MYOD1 (red), confirming their myogenic identity. Nuclei are counterstained with DAPI (blue). Scale bar = 50 μm. **B** SA-β-gal staining in primary myoblasts isolated from young and aged individuals (*n* = 3). Scale bar = 100 μm. **C** Western blot analysis of CTRP9, LAMP2A, and NLRP3 protein expression in young and aged human myoblasts; GAPDH serves as an internal reference (*n* = 3). **D** Quantification of SA-β-gal staining in siNC and siLAMP2A groups (*n* = 3). Scale bar = 100 μm. **E** Western blot analysis of NLRP3 expression following LAMP2A knockdown in human primary myoblasts; GAPDH serves as the loading control (*n* = 3). **F** SA-β-gal staining of aged human myoblasts treated with gCTRP9 (5 μg/mL, 24 h), (*n* = 3). Scale bar = 100 μm. **G** Western blot analysis of LAMP2A and NLRP3 expression in aged human myoblasts treated with gCTRP9; GAPDH serves as the loading control (*n* = 3). Data are presented as mean ± SEM.

To assess the translational relevance of our findings, we isolated primary myoblasts from the gastrocnemius muscles of young (*n* = 3) and elderly (*n* = 3) human donors. Myoblast identity was confirmed by MYOD1 expression (Fig. 8A). SA-β-gal staining revealed a significant increase in senescent cell area in aged myoblasts (Fig. 8B). Western blot analysis showed decreased CTRP9 and LAMP2A levels, accompanied by elevated NLRP3 expression in aged cells, mirroring our murine data (Fig. 8C).

To explore the functional role of LAMP2A, we performed siRNA-mediated knockdown in human myoblasts. LAMP2A silencing led to increased NLRP3 expression and enhanced SA-β-gal staining, indicating augmented senescence (Fig. 8D, E).

Treatment with recombinant gCTRP9 (5 μg/mL, 24 h) significantly reduced senescent cell burden in aged myoblasts (Fig. 8F). This was accompanied by restored LAMP2A expression and reduced NLRP3 levels, as shown by Western blotting (Fig. 8G), confirming that gCTRP9 reactivates CMA and suppresses inflammasome activation in human primary myoblasts.

In a nutshell, these results demonstrate that the CTRP9–LAMP2A–NLRP3 axis is disrupted in aged human myoblasts, and that gCTRP9 can reverse age-related cellular senescence by restoring this pathway.

## Discussion

In this study, we demonstrate for the first time that CTRP9 promotes the degradation of NLRP3 via activation of LAMP2A-mediated chaperone-mediated autophagy (CMA) in sarcopenia, thereby exerting anti-senescent and anti-inflammatory effects. Notably, gCTRP9 appears to regulate several critical aspects of muscle degeneration and regeneration, including (i) attenuation of cellular senescence, (ii) enhancement of CMA activity, (iii) degradation of NLRP3, and (iv) reduction of IL-1β levels. This molecule displays dual functions: anti-atrophy by reducing senescence and inflammatory markers and improving myotube morphology, and pro-differentiation by increasing MYOD1 expression and fusion capacity. These findings highlight a dual regulatory mechanism of CTRP9 that may be of therapeutic value for combating sarcopenia.

We first confirm that CTRP9 is secreted in an autocrine manner in both C2C12 cells and skeletal muscle tissue. Its active form, gCTRP9, significantly decreases the expression of aging-related markers such as p16, p21, and IL-1β, improves myotube morphology, and enhances myogenic differentiation. While few adipomyokines—such as IL-6, FGF21, and irisin—have been shown to regulate these processes, our findings newly position gCTRP9 as a member of this functional category [39].

Mechanistically, gCTRP9 enhances the degradation of NLRP3 through the activation of CMA, thereby preventing excessive inflammasome activation and the release of downstream inflammatory cytokines. As LAMP2A is the rate-limiting component of CMA, its upregulation proves essential for CMA functionality. Our protein–protein docking and co-immunoprecipitation assays confirm a direct physical interaction between CTRP9 and LAMP2A, thereby strengthening the LAMP2A-mediated function. These results collectively emphasize that CTRP9 offers protective effects against both myotube atrophy and impaired differentiation, hallmark features of sarcopenia.

Importantly, we employ a replicative senescence model in C2C12 cells, a model that has been widely used in sarcopenia-related research. This model mimics key features of aging skeletal muscle—namely, myotube thinning and loss of differentiation potential—through serial passaging-induced senescence. Previous studies consistently reported 50–80% reductions in fusion and maturation indices in aged C2C12 cells compared with low-passage controls [32, 33, 40]. We validate this phenotype in our study using SA-β-gal staining, myotube morphology, and fusion index analysis. This well-established model thus provides a robust and reproducible platform for studying the molecular mechanisms underlying muscle aging.

To assess the translational relevance of our findings, we isolated primary human myoblasts from the gastrocnemius muscle of three young and three elderly donors. We observed a significant downregulation of CTRP9 and LAMP2A, along with increased NLRP3 expression in the elderly group, consistent with our in vitro and in vivo results. Although the limited human sample size represents a study limitation, it provides valuable preliminary insight. We suggest that future studies expand this cohort to enhance statistical power and generalizability. Additionally, in our study, due to the absence of a validated siRNA sequence targeting the human CTRP9 CCDS region, CTRP9 knockdown experiments were not performed in human cells. Instead, only LAMP2A silencing was conducted in primary myoblasts, and we aim to address this issue in subsequent work.

During the mechanistic investigation, we observed that gCTRP9 treatment increased LC3B degradation in C2C12 cells, suggesting a potential impact on the autophagy process. However, LC3B expression alone cannot confirm autophagy activation, as LC3 accumulation may also reflect impaired autophagic flux. To clarify this, we employed various autophagy and proteasome inhibitors. Only chloroquine (CQ), a lysosomal inhibitor, was able to reverse gCTRP9-induced NLRP3 degradation, while 3-MA (an autophagy initiation inhibitor) and MG132 (a proteasome inhibitor) had no significant effects. These findings suggest that gCTRP9 acts primarily through a lysosome-dependent, CMA-like pathway rather than canonical macroautophagy or proteasome degradation.

Moreover, given that LAMP2A also contributes to lysosome function and autophagosome–lysosome fusion, we cannot entirely exclude the possibility that CTRP9 indirectly affects macroautophagy via modulation of LAMP2A. Our future studies will employ dynamic autophagic flux assays, such as mCherry-GFP-LC3B tandem reporters, to dissect the role of CTRP9 in various autophagic pathways.

In summary, our study provides some novel insights into the dual anti-atrophic and pro-differentiation roles of CTRP9, mediated via LAMP2A-dependent CMA activation and subsequent NLRP3 degradation. These results confirm CTRP9 as both an anti-inflammatory regulator and a promoter of skeletal muscle regeneration. This dual protective mechanism suggests that targeting the CTRP9-LAMP2A-NLRP3 axis for sarcopenia intervention is a promising therapeutic avenue for treating the disease.

## Supplementary information

Figure S1

Figure S2

Figure S3

Figure S4

Supplementary Table 1

Supplementary Table 2

Supplementary Table 3

Supplementary Table 4

Supplementary legends

Original western blots

## Data Availability

The paper and its supplementary materials contain all necessary data for evaluating the conclusions. Supplementary information is available at Cell Death & Disease’s website.
