# Supplementary figures and images for: CTRP9 as a myokine mitigates sarcopenia via the LAMP-2A/NLRP3 pathway
Source: Cell Death Dis. 2025 Oct 7;16(1):710. doi: 10.1038/s41419-025-08025-w (PMC12504663; doi:10.1038/s41419-025-08025-w)

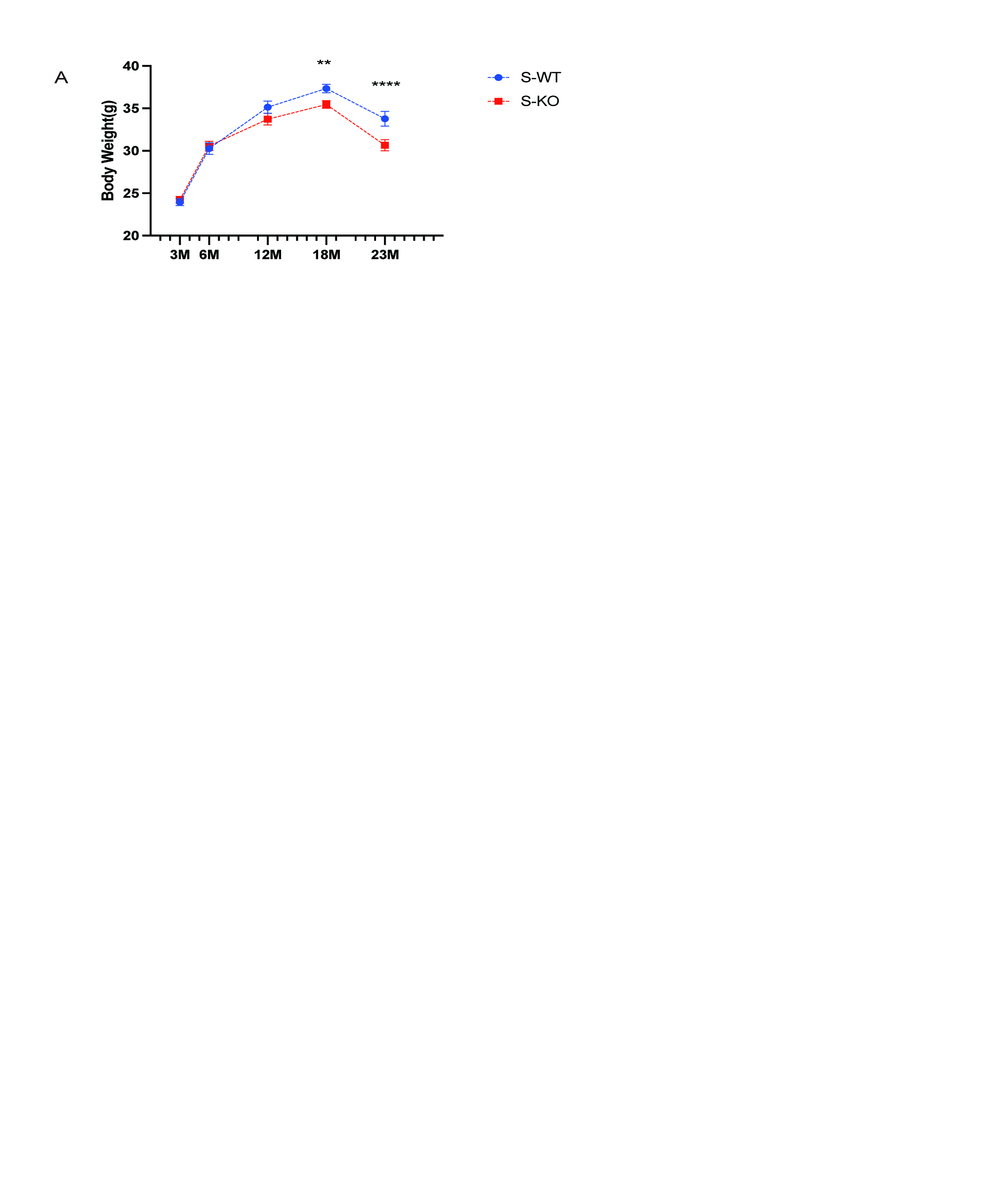

Supplement: Supplementary file 1 — Figure S1 [file 41419_2025_8025_MOESM1_ESM.tif]

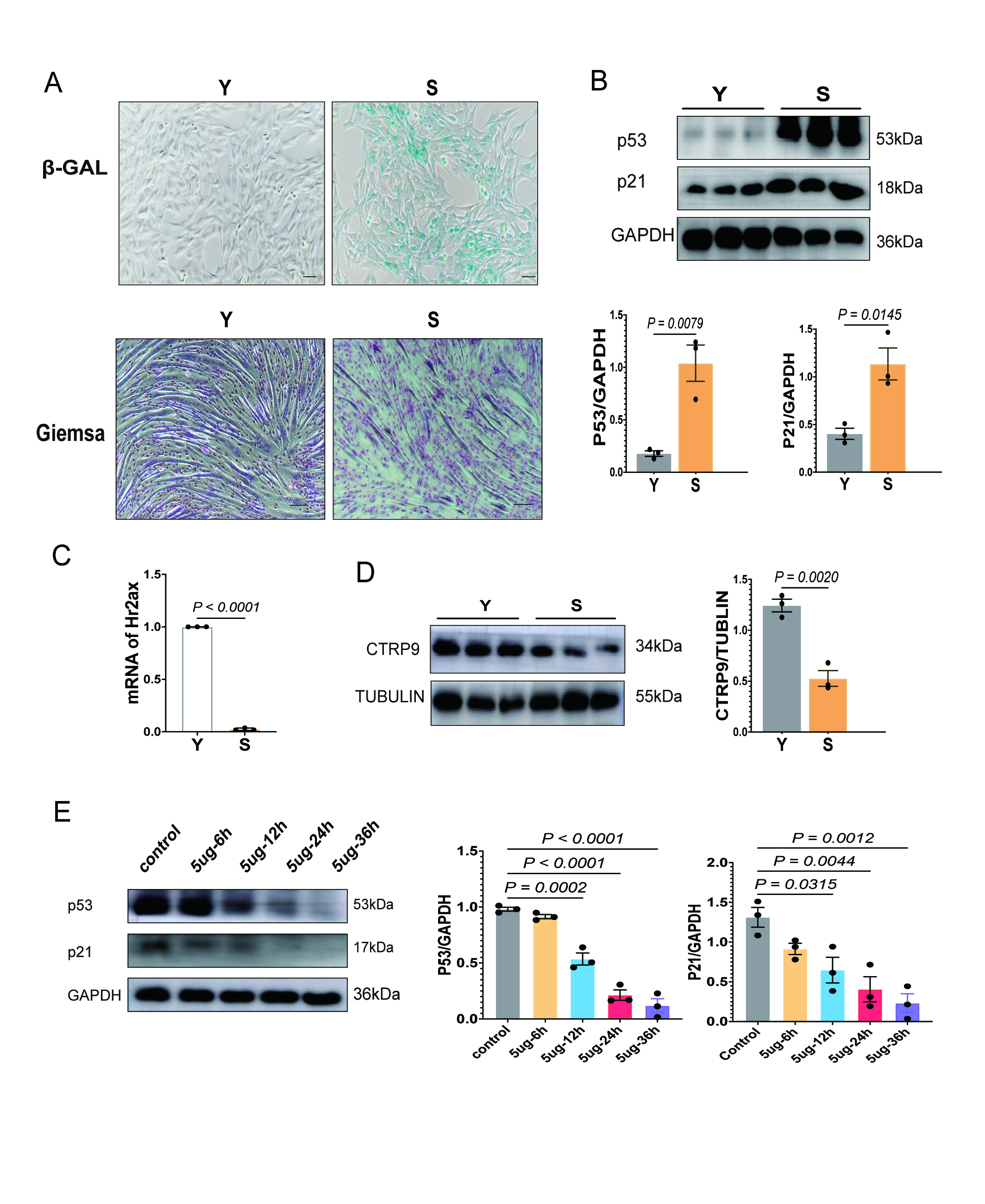

Supplement: Supplementary file 2 — Figure S2 [file 41419_2025_8025_MOESM2_ESM.tif]

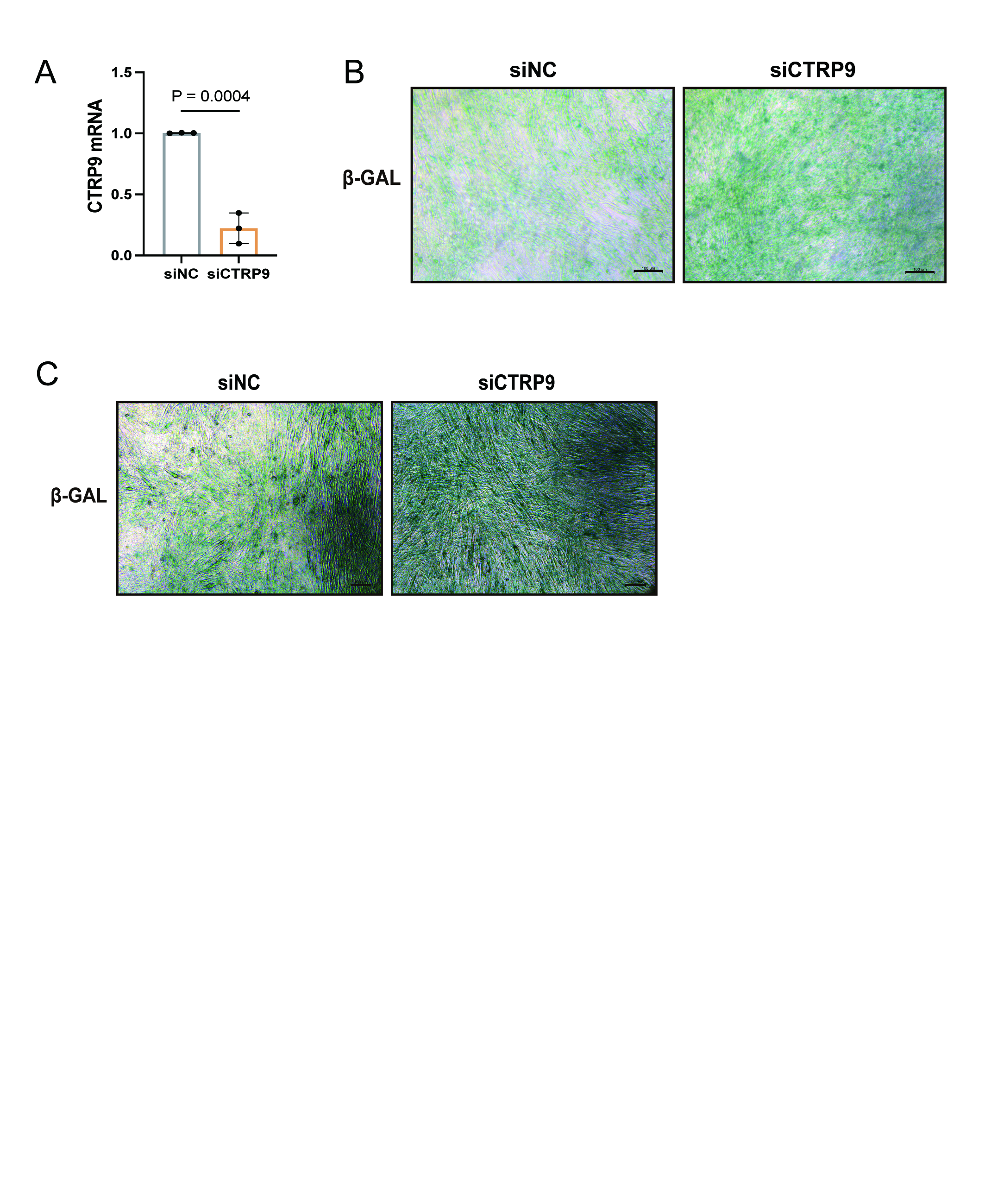

Supplement: Supplementary file 3 — Figure S3 [file 41419_2025_8025_MOESM3_ESM.tif]

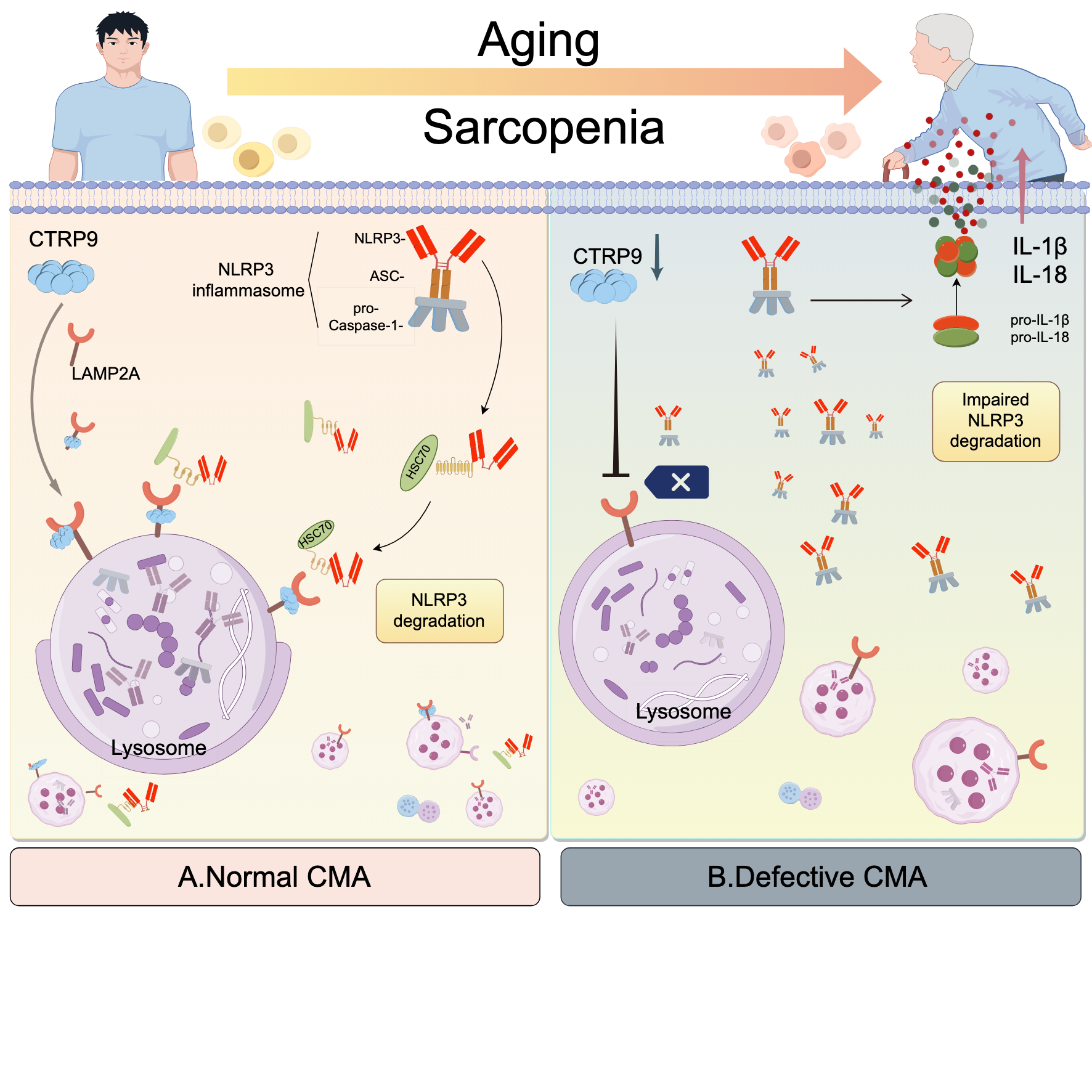

Supplement: Supplementary file 4 — Figure S4 [file 41419_2025_8025_MOESM4_ESM.tif]

1C

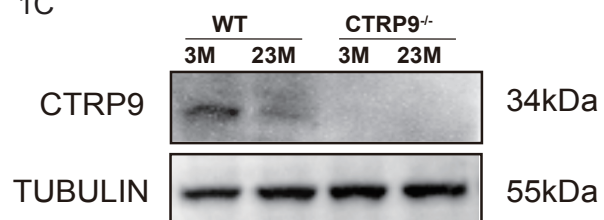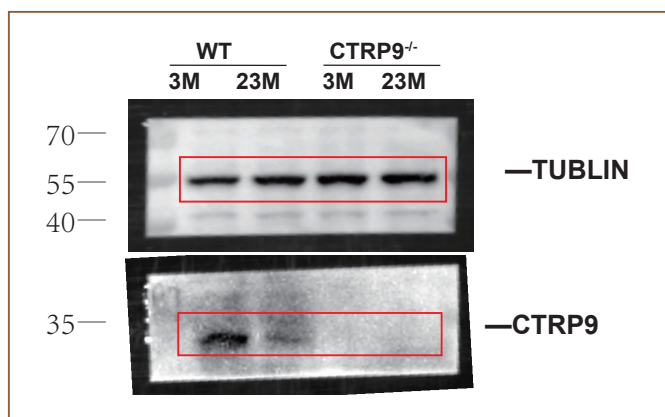

1E

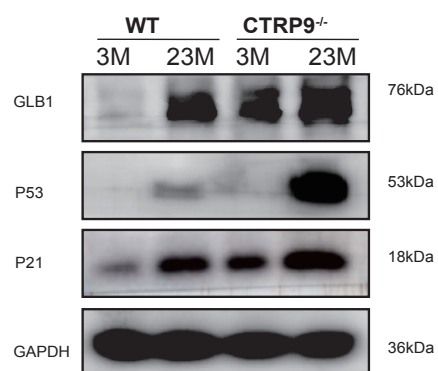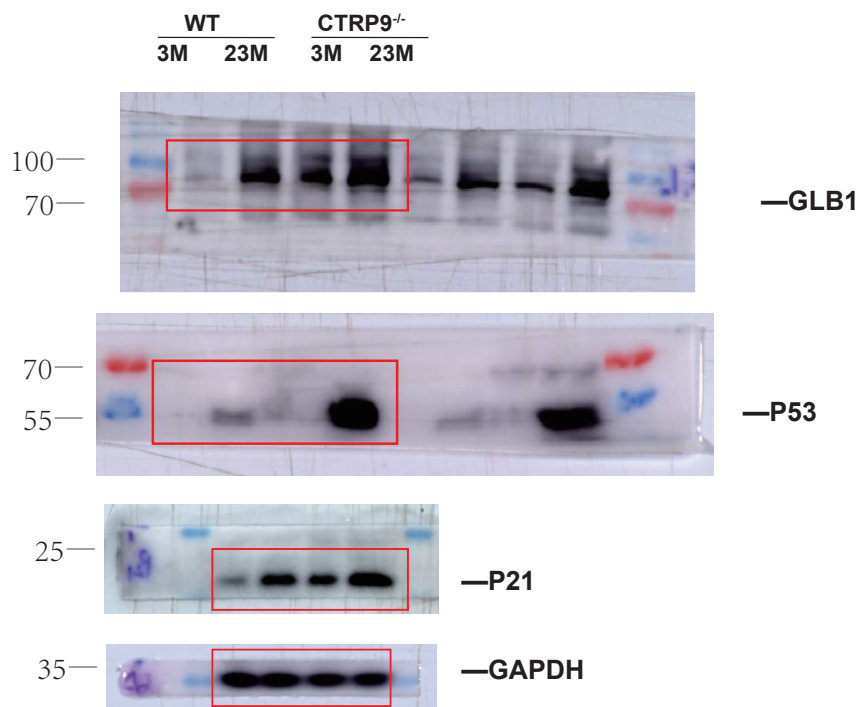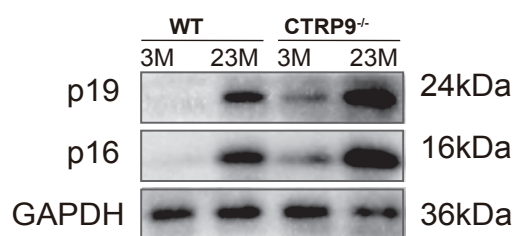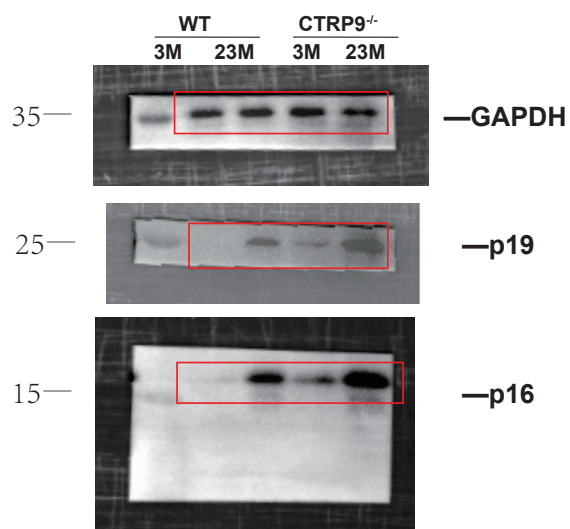

2D

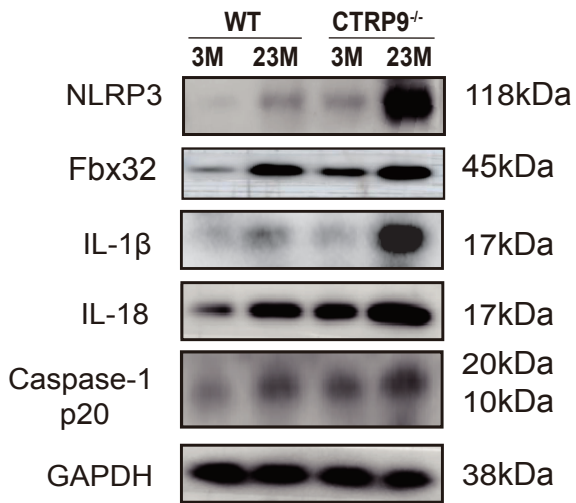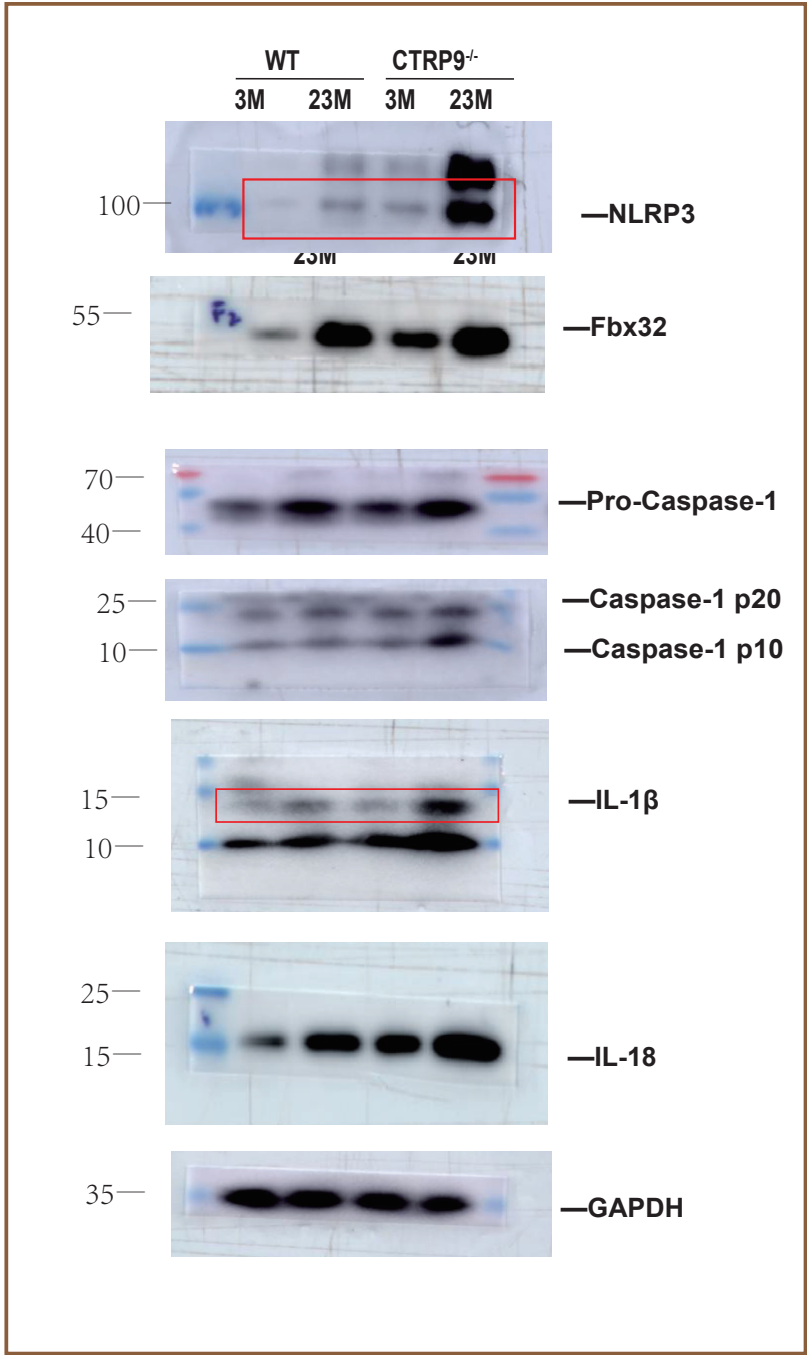

S2B

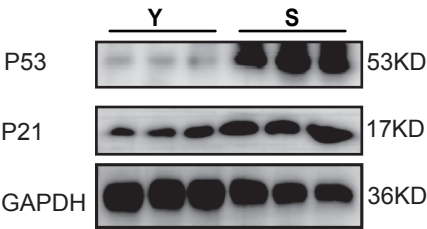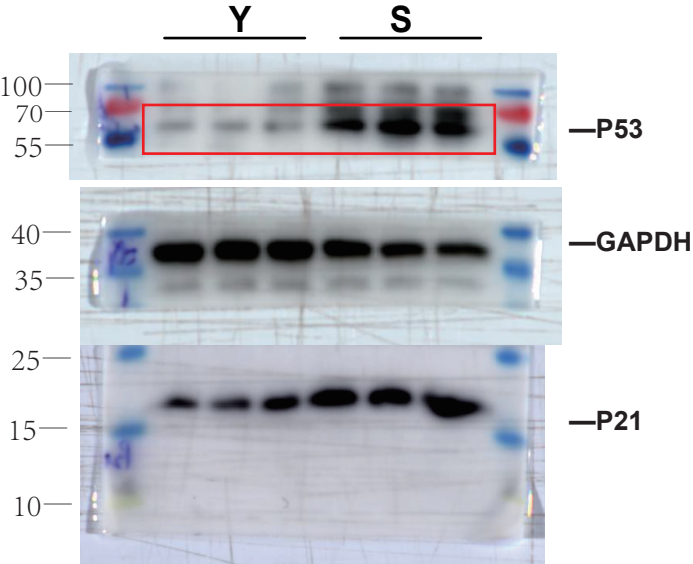

S2C

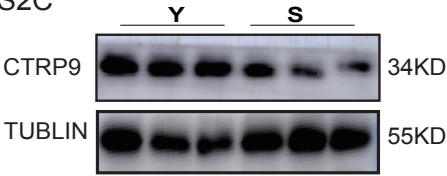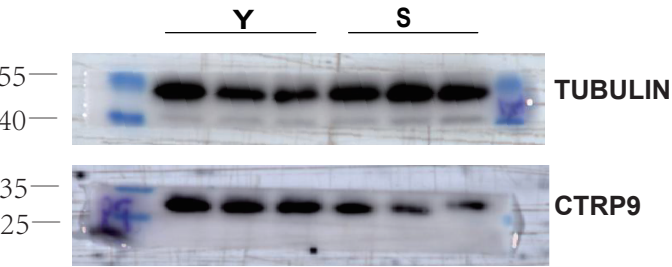

S2F

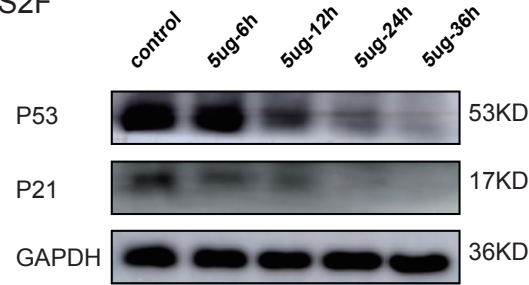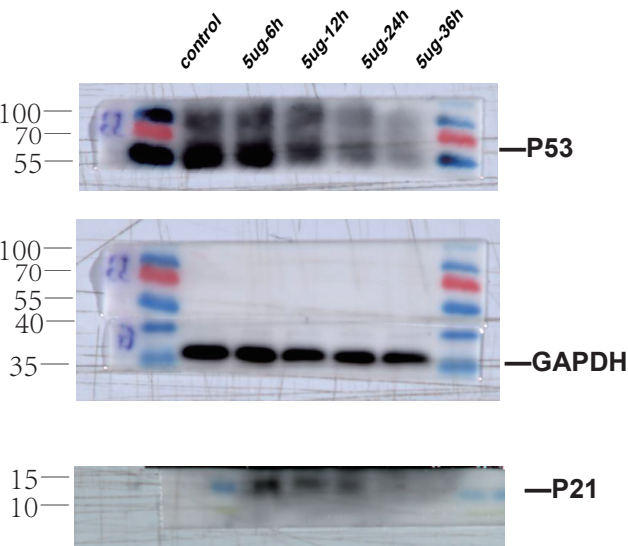

3A

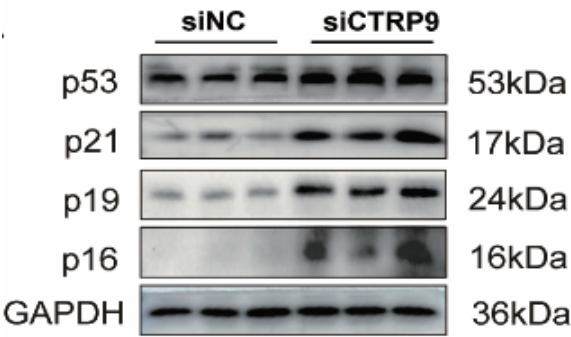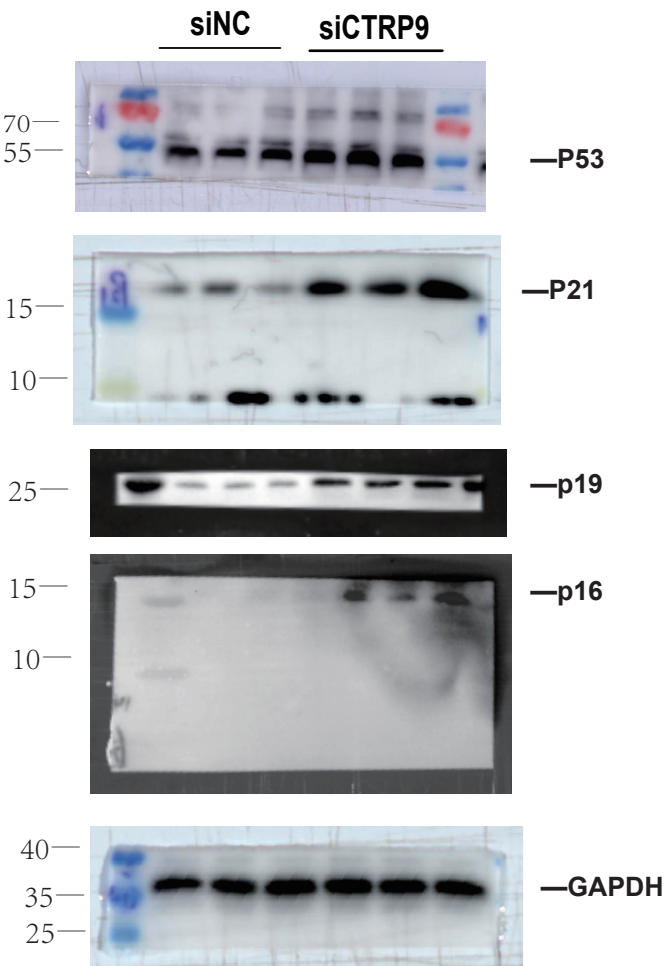

3E

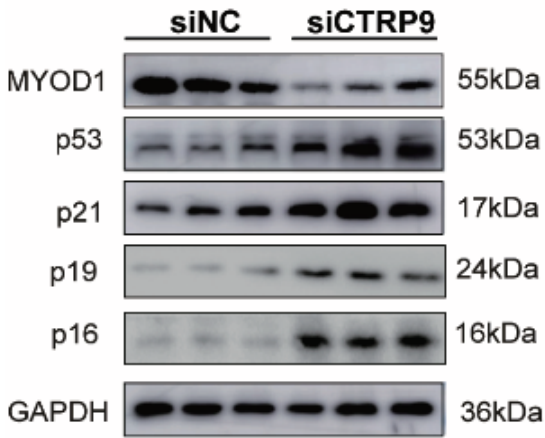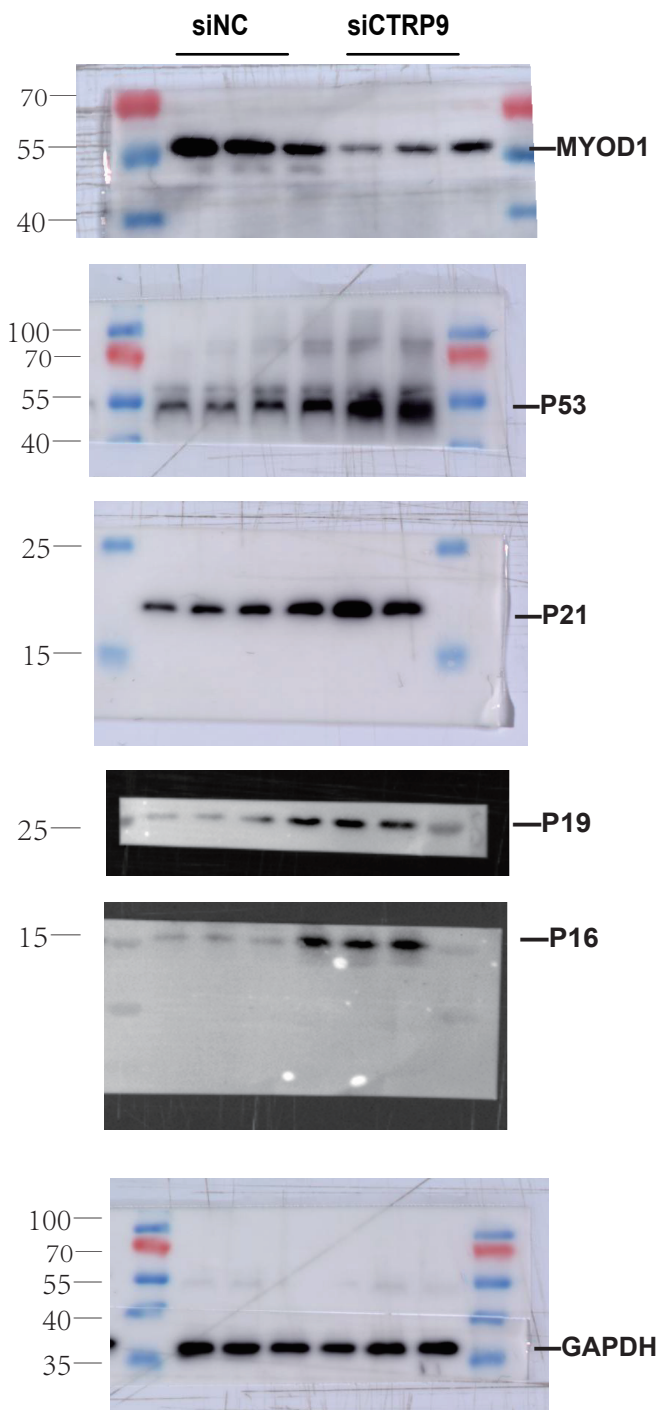

3D

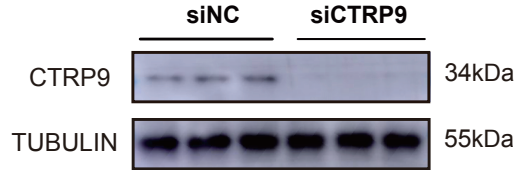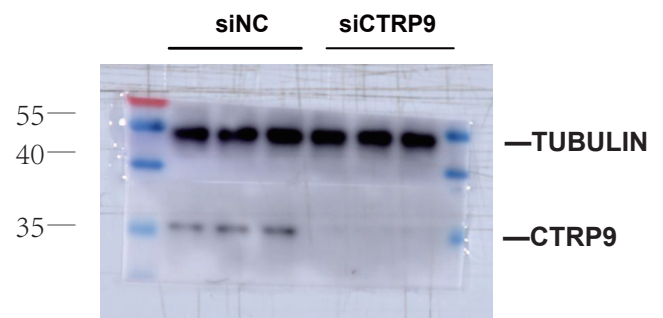

4A

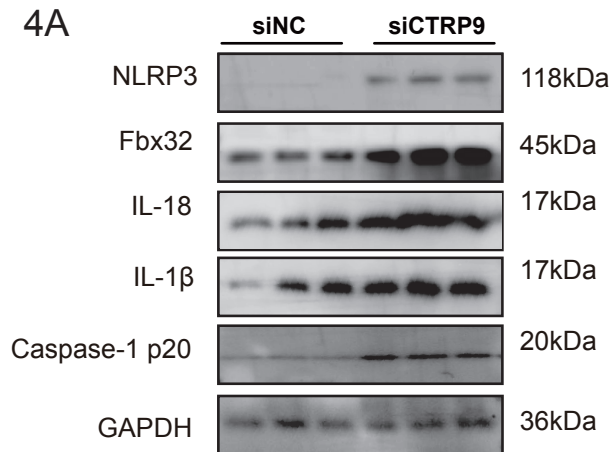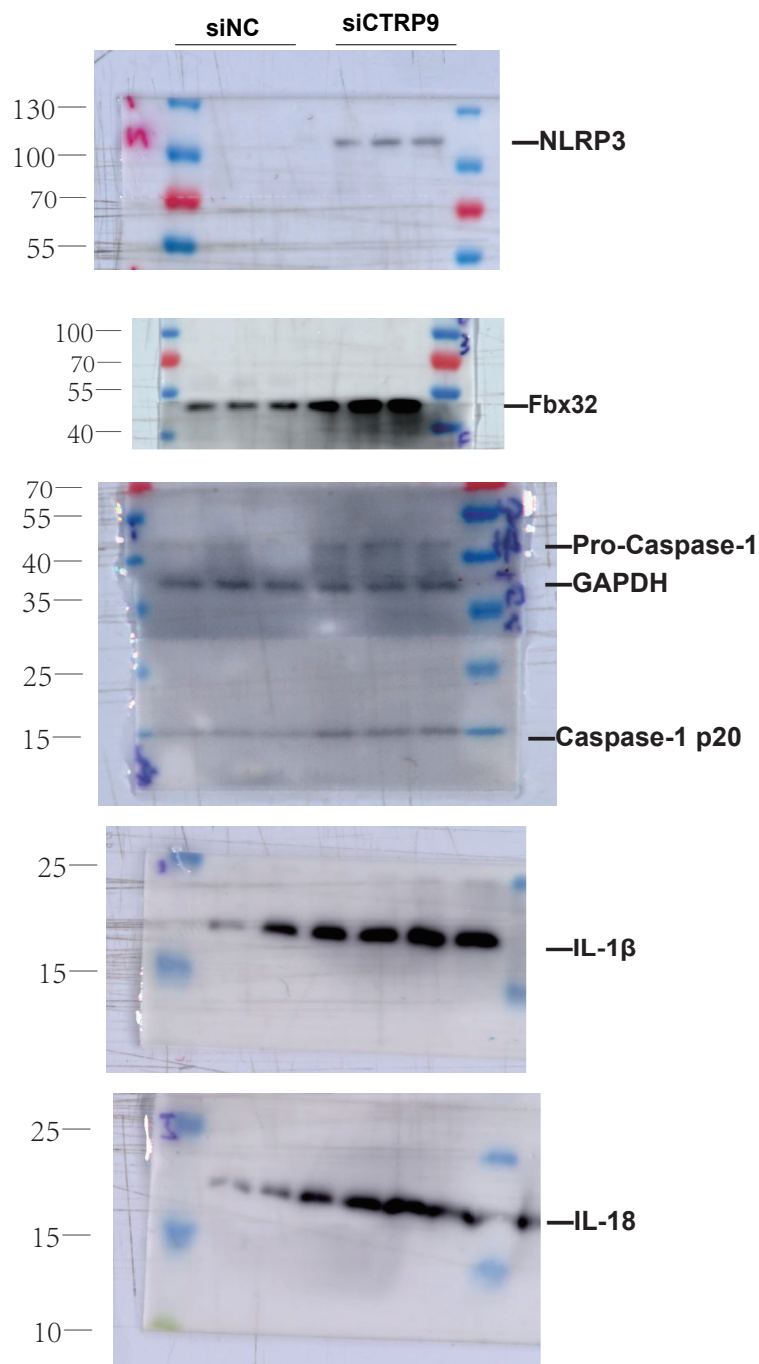

4E

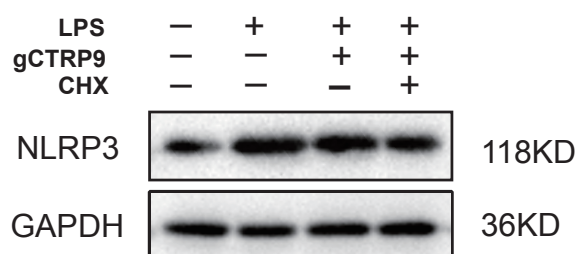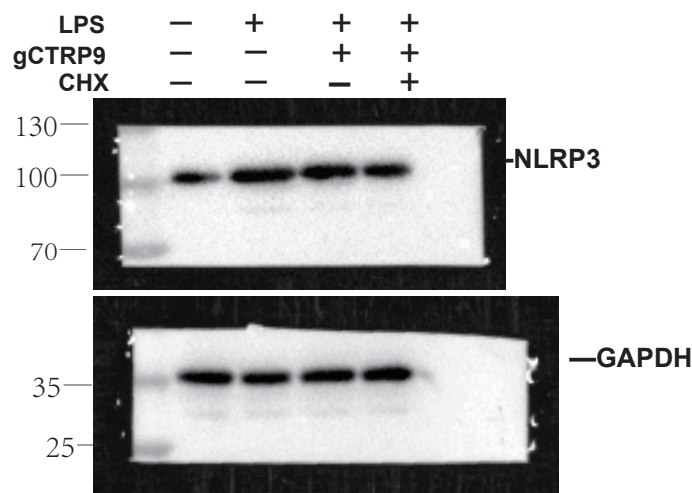

4F

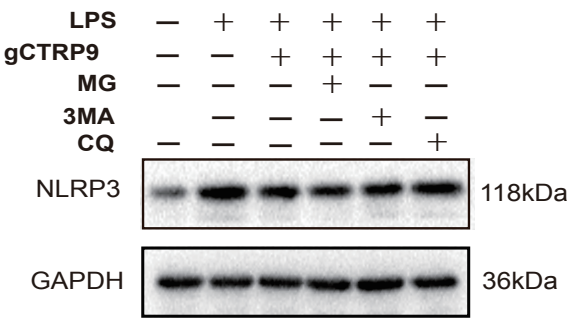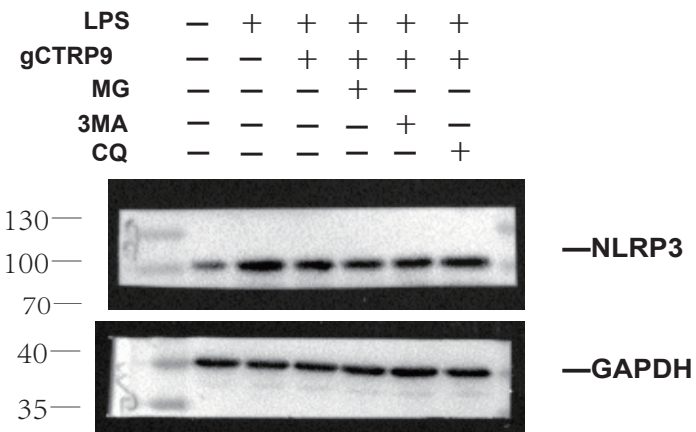

5A

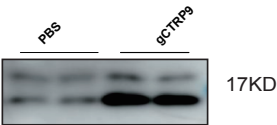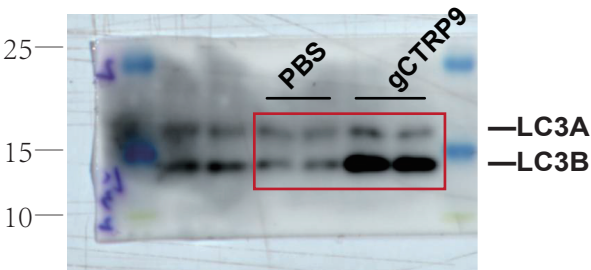

5B

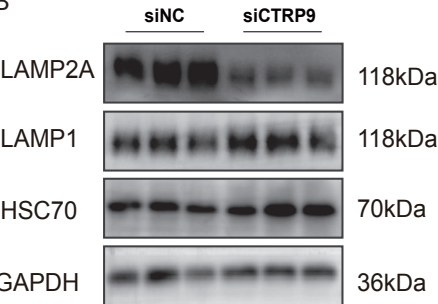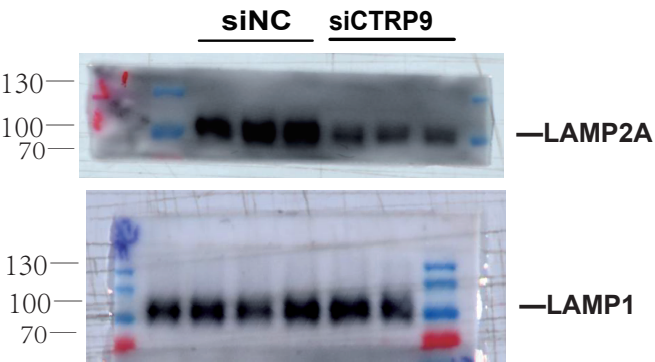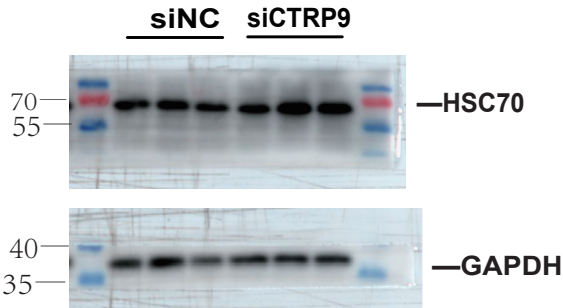

5D

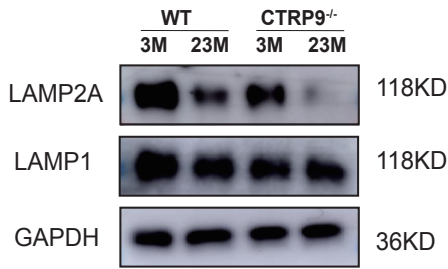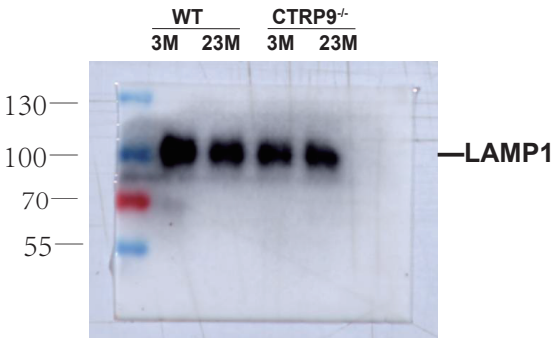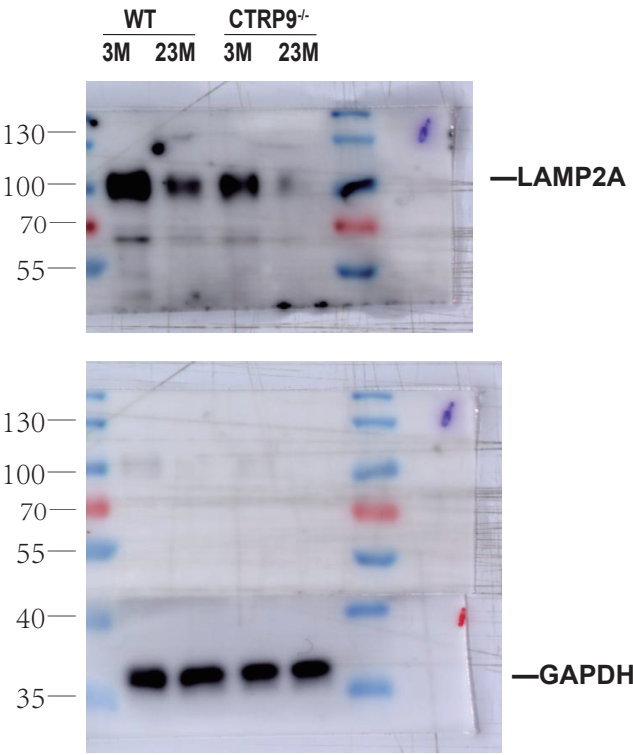

5F

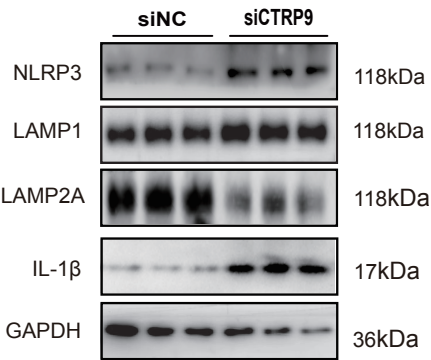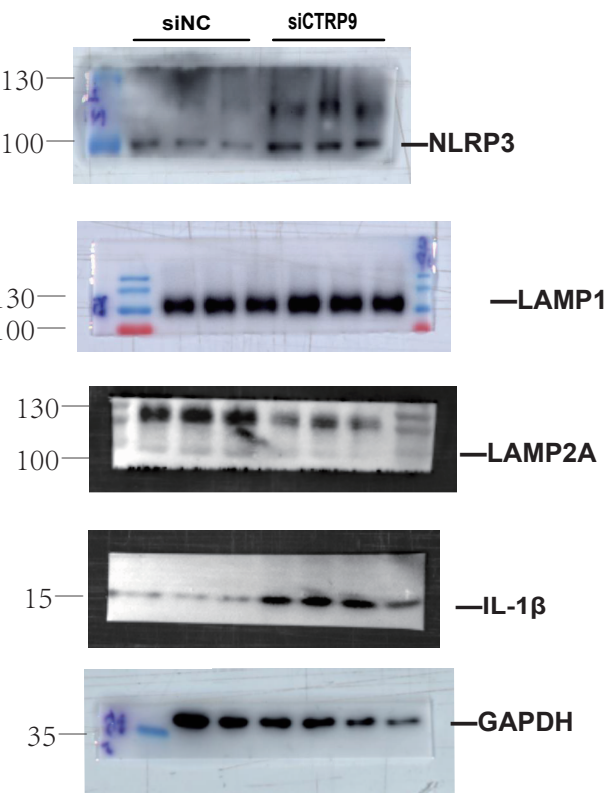

6A

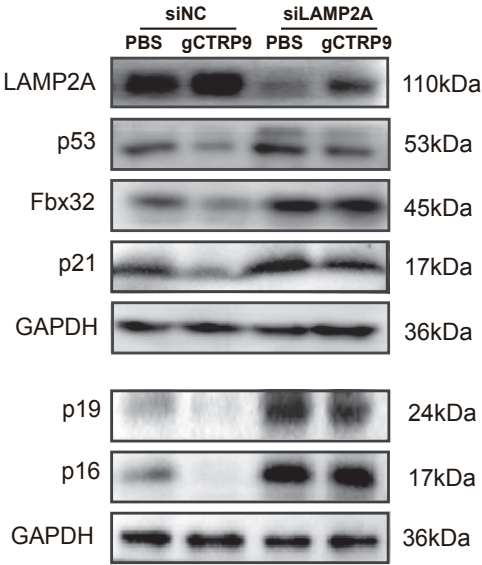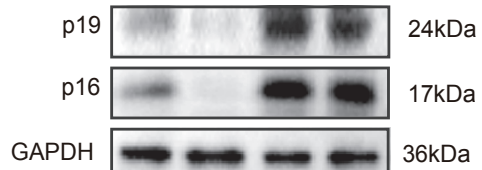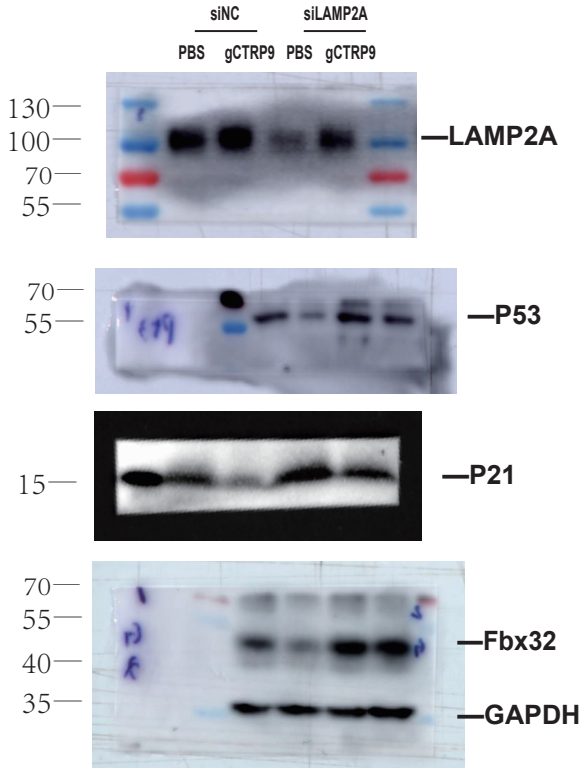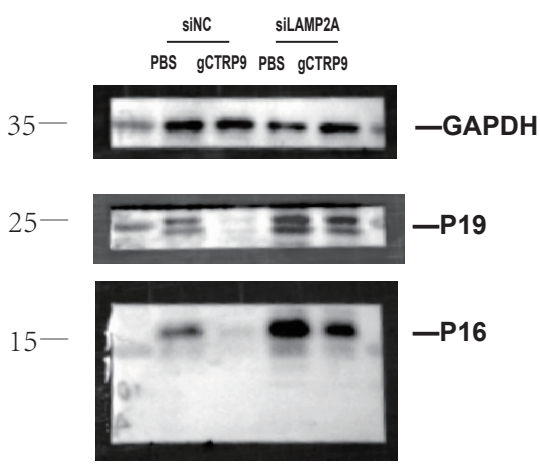

6D

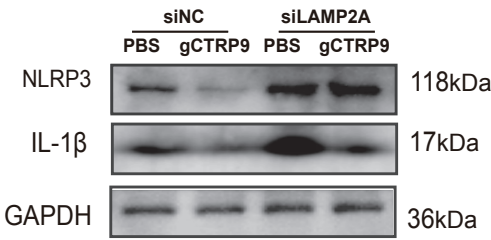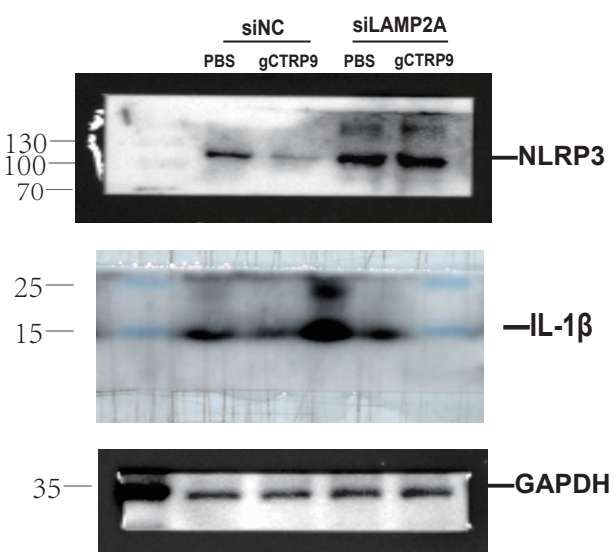

7A

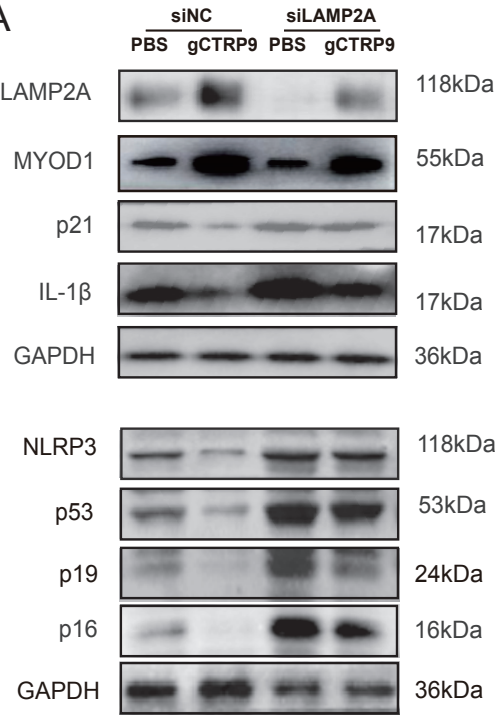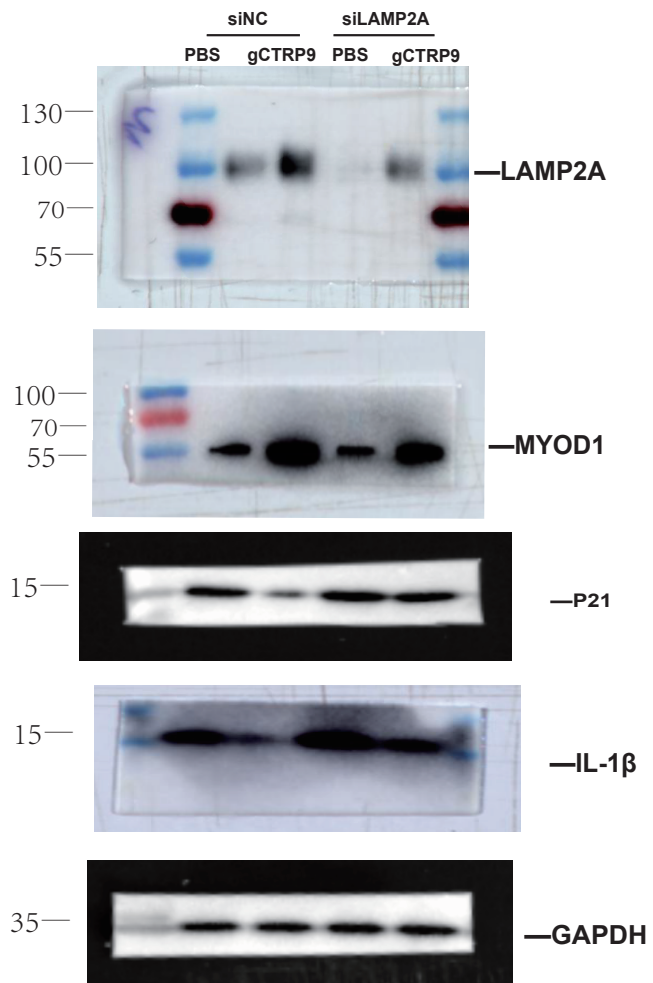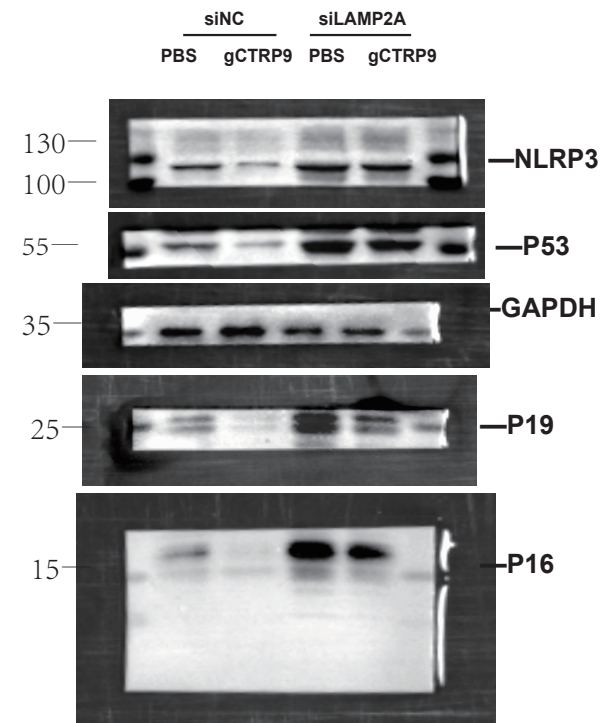

7F

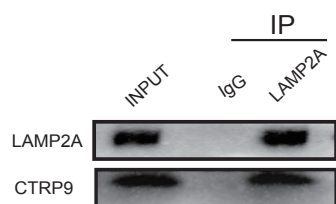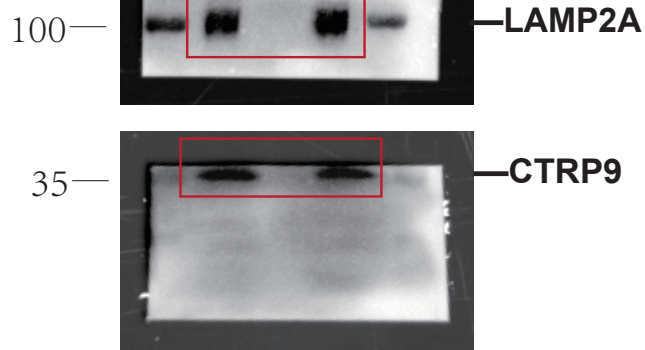

8C

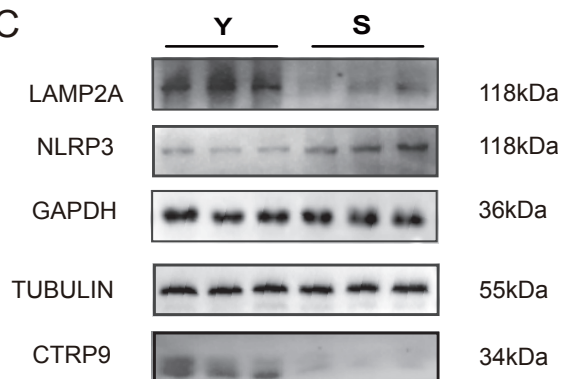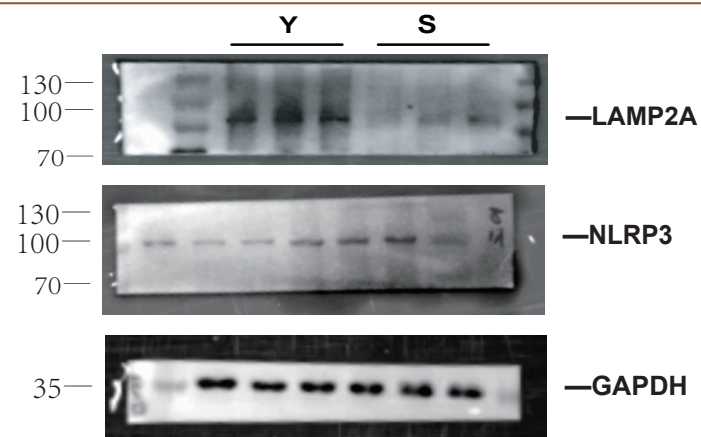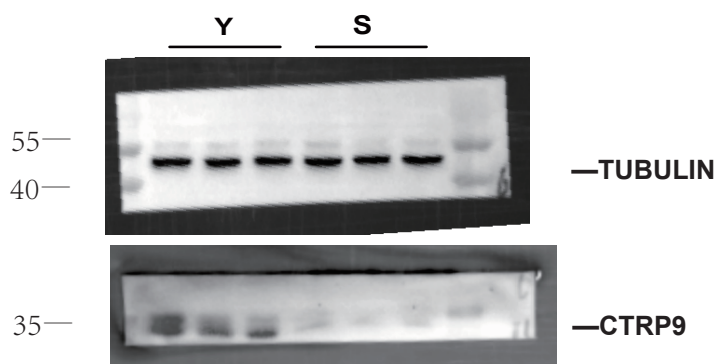

8E

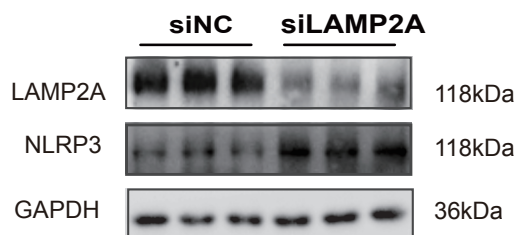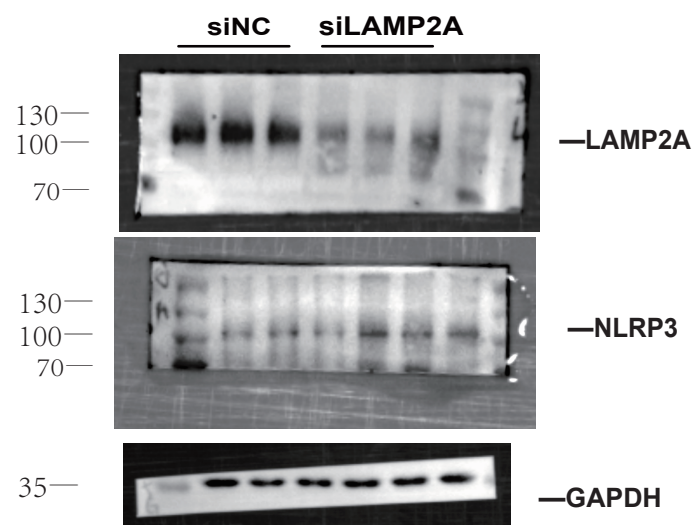



8G

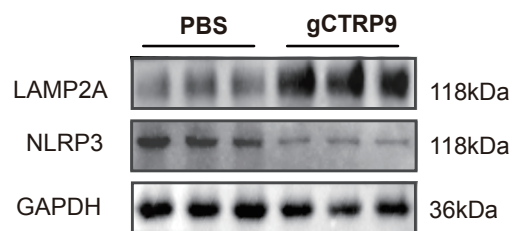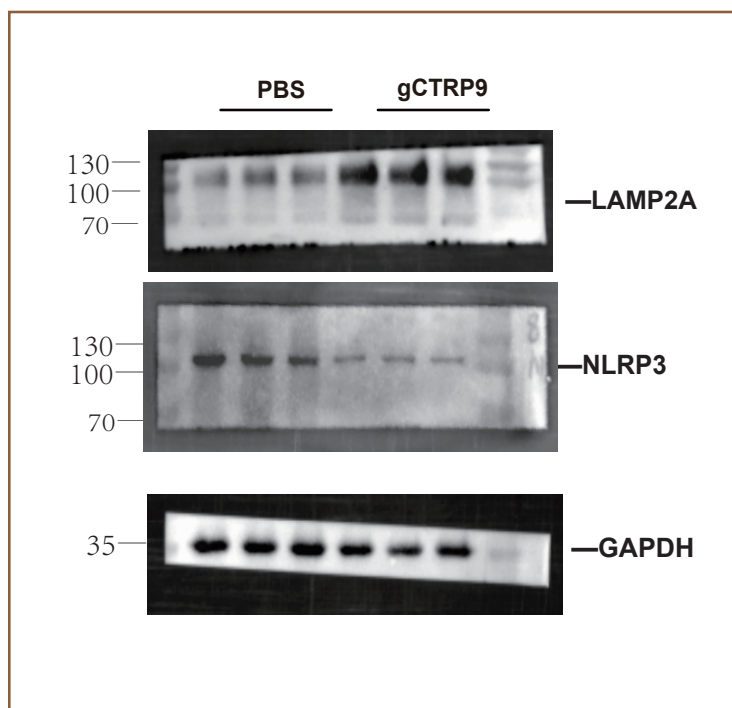

Supplement: Supplementary file 10 — Original western blots [file 41419_2025_8025_MOESM10_ESM.pdf]
