# Supplementary material for: CTRP9 as a myokine mitigates sarcopenia via the LAMP-2A/NLRP3 pathway
Source: Cell Death Dis. 2025 Oct 7;16(1):710. doi: 10.1038/s41419-025-08025-w (PMC12504663; doi:10.1038/s41419-025-08025-w)
Supplement: Supplementary file 9 — Supplementary legends [file 41419_2025_8025_MOESM9_ESM.docx]

**Supplementary Figure.1: CTRP9 Deficiency Results in Reduced Body Weight in Aged Mice.**

**A** Body weight measurements of wild-type (S-WT) and CTRP9 knockout (S-KO) male mice at 3, 6, 12, 18, and 23 months of age (n = 6 per group). S-KO mice exhibited significantly lower body weight at 18 and 23 months compared with age-matched S-WT controls. Data are presented as mean ± SEM. Statistical significance is determined by two-way ANOVA followed by Bonferroni post hoc test (**p< 0.01, ****p < 0.0001).

**Supplementary Figure.2: The Replicative Senescence Model is Successfully Established in C2C12 Cells, Accompanied by Reduced CTRP9 Expression.**

**A** Representative images of SA-β-gal staining and Giemsa staining in early-passage (young) and long-term passaged (replicative senescent) C2C12 myoblasts. Scale bar = 100µm (n = 3). **B** Western blot analysis of p53 and p21 protein levels in young and senescent C2C12 cells; GAPDH serves as the loading control (n = 3).

**C** qPCR analysis of Hr2ax mRNA expression in young and senescent C2C12 cells. (n = 3). **D** Western blot analysis of CTRP9 protein expression in replicative senescent C2C12 cells compared to controls; Tubulin serves as the loading control (n = 3). E. Western blot analysis of p53 and p21 protein expression in replicative senescent C2C12 myoblasts treated with recombinant globular CTRP9 (gCTRP9, 5 µg/mL) for 0, 12, 24, and 36 hours; GAPDH serves as the loading control (n = 3).

Data are presented as mean ± SEM.

**Supplementary Figure .3: CTRP9 Knockdown Accelerates Senescence and atrophy in Differentiated C2C12 Myotubes and Impairs Myogenesis in Replicative Aging C2C12 Myoblasts.**

**A** qPCR analysis of CTRP9 mRNA expression in replicative senescent C2C12 myotubes transfected with siCTRP9 or siNC (n = 3). **B** SA-β-gal staining in C2C12 myotubes transfected with siCTRP9 or siNC (n = 3). Scale bar = 100μm. **C** Representative images of SA-β-gal staining in C2C12 myoblasts transfected with siCTRP9 or siNC (n = 3).

Data are presented as mean ± SEM.

**Supplementary Figure.4: The Present Study Hypothesizes That the Knockdown of CTRP9 Leads to a Mechanism by Which Defects in Chaperone-mediated Autophagy (CMA) Promote the Activation of the NLRP3 (NOD-like Receptor Family, Pyrin Domain Containing 3) Inflammasome.**

**A** In C2C12 cells with normal CMA activity, CTRP9 facilitated the degradation of NLRP3 proteins through the LAMP2A-mediated CMA pathway, thereby preventing overactivation of the NLRP3 inflammasome. **B** In C2C12 cells with defective CTRP9, NLRP3 proteins were not efficiently degraded or cleared, leading to the overactivation of the NLRP3 inflammasome. This overactivation resulted in the cleavage of pro-IL-1β and pro-IL-18 into their mature forms, IL-1β and IL-18, respectively, thereby contributing to sarcopenia.

Abbreviations: ASC indicates apoptosis-associated speck-like protein containing a CARD (C-terminal Caspase-recruitment domain); HSC70 refers to heat shock cognate 71kDa protein.

*Created with Figdraw (*[*www.figdraw.com*](http://www.figdraw.com)*).*

### **Supplementary Tab. 1: Baseline Characteristics of the Included Participants.**

**Abbreviations:** LDL-C, low-density lipoprotein cholesterol; HDL-C, high-density lipoprotein cholesterol; TG, triglyceride; TC, total cholesterol; Cr, serum creatinine; AST, aspartate transaminase; ALT, alanine aminotransferase; BUN, blood urea nitrogen; Glucose, blood glucose.Data are shown as mean ± SEM or percentage (%) of subjects in each group.  Statistical significance using c χ2 test for categorical data and done-way ANOVA test for continuous data followed by Bonferroni correction.

Data are presented as mean ± SD, median (25th–75th percentile), or percentage (%) of participants in each group. Statistical significance was assessed using the χ² test for categorical variables and one-way ANOVA for continuous variables, followed by Bonferroni post hoc correction.

**Supplementary Tab.S2: siRNA sequences used in this study.**

**Supplementary Tab.S3: Primer sequences used for qRT-PCR.**

**Supplementary Tab.S4: Antibody Validation.**
